# Comparative Analysis of the *pIgR* Gene from the Antarctic Teleost *Trematomus bernacchii* Reveals Distinctive Features of Cold-Adapted Notothenioidei

**DOI:** 10.3390/ijms23147783

**Published:** 2022-07-14

**Authors:** Alessia Ametrano, Simona Picchietti, Laura Guerra, Stefano Giacomelli, Umberto Oreste, Maria Rosaria Coscia

**Affiliations:** 1 Institute of Biochemistry and Cell Biology, National Research Council of Italy, Via P. Castellino 111, 80131 Naples, Italy; alessia.ametrano@ibbc.cnr.it (A.A.); stefano77g@hotmail.it (S.G.); umberto.oreste@ibbc.cnr.it (U.O.); 2 Department for Innovation in Biological, Agro-Food and Forest Systems, University of Tuscia, Largo dell’Università snc, 01100 Viterbo, Italy; picchietti@unitus.it (S.P.); lauraguerra@unitus.it (L.G.)

**Keywords:** pIgR, gene structure, cold environment, gene expression, teleost immunity, adaptive evolution, mucosal tissues, genome alteration, Notothenioidei, IgV domains

## Abstract

The IgM and IgT classes were previously identified and characterized in the Antarctic teleost *Trematomus bernacchii*, a species belonging to the Perciform suborder Notothenoidei. Herein, we characterized the gene encoding the polymeric immunoglobulin receptor (pIgR) in the same species and compared it to the pIgR of multiple teleost species belonging to five perciform suborders, including 11 Antarctic and 1 non-Antarctic (*Cottoperca gobio*) notothenioid species, the latter living in the less-cold peri-Antarctic sea. Antarctic *pIgR* genes displayed particularly long introns marked by sites of transposable elements and transcription factors. Furthermore, analysis of *T. bernacchii* pIgR cDNA unveiled multiple amino acid substitutions unique to the Antarctic species, all introducing adaptive features, including N-glycosylation sequons. Interestingly, *C. gobio* shared most features with the other perciforms rather than with the cold-adapted relatives. *T. bernacchii* pIgR transcripts were predominantly expressed in mucosal tissues, as indicated by q-PCR and in situ hybridization analysis. These results suggest that in cold-adapted species, pIgR preserved its fundamental role in mucosal immune defense, although remarkable gene structure modifications occurred.

## 1. Introduction

The polymeric immunoglobulin receptor (pIgR) appeared early during evolution in teleost fishes [1] and coevolved with mucosal Ig isotypes, ensuring mucosal protection [2]. It has a conserved structure, consisting of an extracellular region composed of varying numbers of immunoglobulin variable (IgV) domains increasing across the evolutionary scale [3], a transmembrane region, and a cytoplasmic tail [4]. In mammals, pIgR has five IgV domains (D), D1–D5, except for bovine and rabbit pIgRs, which only have three of the five domains, D1, D4, and D5, derived from alternative splicing [5,6]. In birds, reptiles, and amphibians, pIgR comprises four domains corresponding to mammalian D1, D3, D4, and D5, respectively [7,8,9]. Fish pIgR shows the simplest topology, comprising only two Ig-like domains, which are homologous to mammalian D1 and D5, based on comparative sequence analyses [10]. Teleost pIgR is known to be expressed in mucosa-associated lymphoid tissues, e.g., the intestine, gills, skin, buccal and pharyngeal cavity, and olfactory system [11]. pIgR has been shown to bind both IgM and IgT, although the exact Ig-binding site of pIgR has not yet been clarified, given that polymeric Igs are devoid of a J chain, contrary to cartilaginous fish, in which the J chain has been identified [12].

Over recent years, great attention has been paid to the function of fish pIgR, while studies aimed at examining its gene structure are still limited. Full-length transcripts from the *pIgR* gene were characterized in various teleost species belonging to different orders [11], and the binding sites of cytokine-inducible regulatory elements, well-known in mammals, were also predicted and considered as potential regulators of the transcription of *pIgR* in teleost fish [13,14,15].

At present, no data are available on pIgR from teleost species living under extreme conditions, such as Notothenioidei (Perciform suborder), which represent the prevalent component of the Antarctic fish fauna. During their evolutionary history, Nototheniodei have undergone extraordinary challenges in adapting to the constantly cold marine environment of Antarctica. The Antarctic notothenioid families have been proposed as a superfamily, named Cryonotethenioidea [16], to be distinguished from the non-Antarctic families that are considered the most phyletically basal branch, having remained in peri-Antarctic seawaters under temperate conditions [17,18]. This taxonomic group has long been considered an attractive model to study biochemical, physiological, and morphological adaptations, although poorly investigated at a molecular level. In previous studies, we investigated the genes encoding IgM and IgT isotypes in several cold-adapted and temperate notothenioid species [19,20,21] and highlighted the first evidence of a possible hepato-biliary transport of Ig in the Antarctic species *Trematomus bernacchii* [22]. 

The most recent advances in collecting omics data from notothenioid fishes provided a source of fundamental information about molecular and genetic features, allowing evolutionary studies on notothenioids in comparison with other perciform species. Thus, the main goal of this study is to investigate the specificities of the *pIgR* gene related to evolutionary adaptation, through a comparative analysis, based on the genomes and transcriptomes available for *T. bernacchii* and 11 Antarctic species belonging to the same suborder as *T. bernacchii* (Notothenoidei). In particular, we extended the analysis to *Trematomus loenbergii*, *Dissostichus eleginoides*, *Dissostichus mawsoni*, *Notothenia coriiceps* (Nototheniidae family), *Harpagifer antarcticus* (Harpagiferidae family), *Gymnodraco acuticeps* (Bathydraconidae family), *Pseudochaenichthys georgianus*, *Chaenocephalus aceratus*, *Chionodraco myersi*, and *Chionodraco hamatus* (Channichthyidae family), all adapted to live in the extreme environment of Antarctica. Moreover, another notothenioid species, *Cottoperca gobio*, belonging to the Bovichtidae family, the ancestral notothenioid family living in more temperate peri-Antarctic seawaters, was added for comparison. Additionally, 26 perciform species belonging to five different families were included for comparative analysis. Finally, the expression of the *T. bernachii pIgR* gene was evaluated through q-PCR and in situ hybridization (ISH), which allowed transcript localization. 

Taken together, these findings underline several peculiar features that may be considered the hallmarks of cold pIgRs and underpin the primary role of pIgR in mucosal immune response and host protection in a cold-adapted teleost species.

## 2. Results

### 2.1. Analysis of T. bernacchii pIgR Gene Locus 

The whole *pIgR* genomic sequence (8310 nt) was retrieved as a single-copy gene from the *T. bernacchii* genome (GenBank assembly accession: NW_022987689) by using NCBI Genome Data Viewer. The *pIgR* gene structure consists of eight exons, interrupted by seven introns. The first exon includes the 5′ UTR and encodes the leader peptide (245 nt); the second exon (345 nt) encodes the D1 domain; the third exon (82 nt) and the 5′ 208-nt of the fourth exon encode the D2 domain; the 3′ end of the fourth (19 nt) exon, the fifth (88 nt) exon, and the 5′-most first end of the sixth (10 nt) exon encode the Extracellular-Membrane Proximal Domain (EMPD); the 5′ end of the sixth exon (51 nt) encodes the transmembrane domain (TM); the 3′ end of the sixth exon (31 nt), along with the seventh (50 nt) and eighth (81 nt) exons, encode the cytoplasmic region. The terminal sequence is 348 nt, including the stop codon (Figure S1). Along with those found in the 3′ UTR, an alternative polyadenylation signal was identified in the fourth intron, and its functionality was assessed. Although this additional site was predicted to have a low confidence score compared to that of the other two, it might be inferred that there is a possible involvement in the transcription of the messenger RNA encoding the secreted form of pIgR. Regarding the gene locus organization, the *T. bernacchii pIgR* gene is flanked by the *dad1* (5383 nt upstream) and *lrrc24* (21,790 nt downstream) genes, as shown in zebrafish and other teleost species [15]. In addition to the *pIgR* gene, a further *pIgR*-*like* gene of 6481 nt was identified in another genomic scaffold (NW_022988066.1). To investigate the evolution of the *pIgR* gene locus, we performed a comparative analysis by considering two Antarctic species belonging to the same Notothenoidei suborder as *T. bernacchii* (Notothenidae family), *G. acuticeps* (Bathydraconidae family), and *P. georgianus* (Channichthyidae family), as well as the non-Antarctic species, *C. gobio* (Bovichtidae family), the latter living in more temperate peri-Antarctic seawater. This analysis was possible due to the considerable amount of sequencing data obtained from the Antarctic notothenioid fish genomes project at the Sanger Institute, which aimed at deepening the very relevant topic of molecular adaptations to extreme conditions.

To identify specific features of the notothenioid *pIgR* gene, we searched databases for homologous sequences from multiple temperate species belonging to other perciform suborders. Twenty-six perciform species belonging to five different suborders and used for comparative analysis are indicated in Table S5 (also see the Section 4). As a first step in the characterization of the *pIgR* gene locus, we investigated the intronic regions. The second and third introns were significantly longer in *T. bernacchii* as well as in the other two Antarctic species compared to those of the temperate species considered, ranging from 2372 to 3080 nt and from 538 to 552 nt, respectively (Table 1). Interestingly, in the case of the non-Antarctic notothenioid *C. gobio*, we observed that the size of the third intron was in line with that of temperate species. Conversely, the other introns were found to be conserved in length across the species considered (Table 1).

Thus, the larger introns accounted for the larger size of the *pIgR* gene of *T. bernacchii*, as well as the other two Antarctic species analyzed.

**Figure 1:**
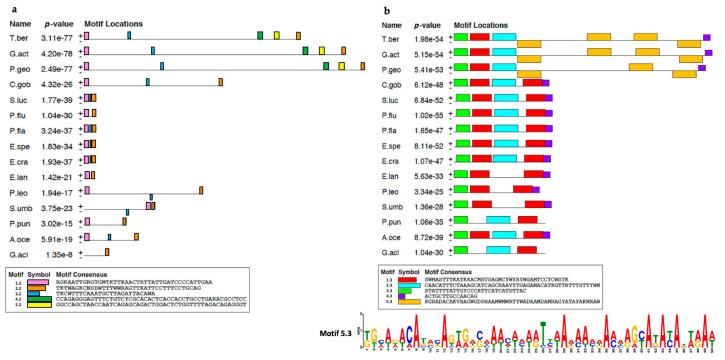
Conserved sequence motifs (colored boxes) identified by MEME in the second (**a**) and third introns (**b**) of *pIgR* from *Trematomus bernacchii* (T.ber), *Gymnodraco acuticeps* (G.act), *Pseudochaennichthys georgianus* (P.geo), *Cottoperca gobio* (C.gob), *Sander lucioperca* (S.luc), *Perca fluviatilis* (P.flu), *Perca flavesvens* (P.fla), *Etheostoma spectabile* (E.spe), *Etheostoma cragini* (E.cra), *Epinephelus lanceolatus* (E.lan), *Plectropomus leopardus* (P.leo), *Sebastes umbrosus* (S.umb), *Pungitius pungitius* (P.pun), *Anarrhichthys ocellatus* (A.oce), and *Gasterosteus aculeatus* (G.acl). The WebLogo representation of motif 5.3 (ocher box) is reported at the bottom of panel (**b**).

Given that the *T. bernacchii* pIgR gene carried extraordinary long introns, factors possibly accounting for this modification were searched using the MEME tool, which allows the identification of conserved motifs repeatedly occurring in a sequence dataset. A comparative analysis conducted on each of the seven introns from notothenioid and temperate species allowed the identification of distinct conserved sequence motifs in Antarctic species (Figure 1). In particular, we found two regions, named motif 4.2 (50 nt) and motif 5.2 (48 nt), exclusively at the 5′ end of the second intron of Antarctic *pIgR* (Figure 1a). Other motifs (motif 1.3, motif 2.3, motif 3.3, and motif 4.3), found in the third intron, were shared by most species, including notothenioids (Figure 1b). Of note, motif 5.3, 50 nt in length, repeated in tandem on both DNA strands of the third intron, represented a distinctive feature of Antarctic fish, as both were absent in the non-Antarctic relative *C. gobio* and in all the other perciforms analyzed. 

**Figure 2:**
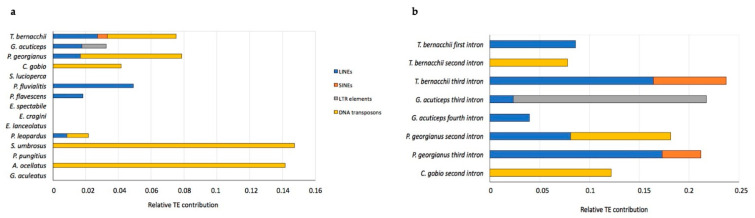
Presence of transposable elements (TEs) in the *pIgR* gene from notothenioids and other perciform species. (**a**) Distribution of TEs in overall pIgR intronic sequences of representatives of each perciform suborder; (**b**) distribution of TEs found in each of the first four *pIgR* introns of Antarctic and non-Antarctic species.

To elucidate other reasons to account for the presence of the very long intronic regions of *pIgR* in *T. bernacchii* and in the other Antarctic species, we searched for transposable elements (TEs), which are known to cause genome modifications. A systemic analysis of the distribution of TE elements in each intron in all Perciformes indicated that the second intron in *T. bernacchii* was characterized by the presence of one type of TE (Figure 2a), while the third intron appeared to be more heterogeneous due to the presence of SINEs and LINEs, the same as found in *P. georgianus*, or similarly to *G. acuticeps*, which showed LINEs and LTR elements (Figure 2b). Moreover, only the second intron of the *C. gobio pIgR* gene was characterized by the presence of DNA transposons, like the *T. bernacchii* second intron (Figure 2a).

To assess whether such long intronic sequences found in the *T. bernacchii pIgR* gene could influence its regulation, we investigated the presence of regulatory signals in the 5′ flanking region and in all introns. Interestingly, in the second and third introns, along with the 5′ flanking region, we identified several putative transcription factor binding sites sharing a very high score (up to 12), with many of them involved in innate immune response, e.g., IL-10 and IFN (Table S1). Similar results were obtained for the other two Antarctic species, *G. acuticeps* and *P. georgianus*. No statistically significant sites were found in the fourth intron either of the notothenioids or in the intronic regions of the temperate species, including the non-Antarctic species. 

**Figure 3:**
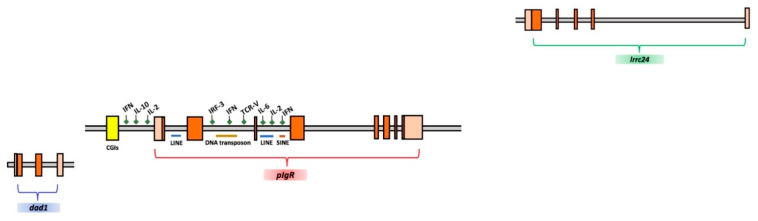
Schematic representation of the organization of the *pIgR* gene locus in *T. bernacchii*. Exons are indicated in orange boxes, promoter and terminal sequences are indicated in bright orange, and the CpG islands (CGIs) are indicated in yellow. Putative transcription factor binding sites (green diamonds) and TEs (blue, ocher, and brown lines) are indicated above and below the scheme, respectively. *dad1* and *lrrc24* flanking genes are also reported.

Of special interest was the search of CpGs islands (CGIs), known as indicators of transcription-promoter sequences. Using the UCSC Genome Browser on *T. bernacchii* and on the two other Antarctic species assemblies, we detected a CGI with lengths ranging from 210 to 348 nt, harboring 21–35 CpGs, located about 500 nt upstream of the 5′ flanking region of the *pIgR* gene (Figure S1). We found that the ObsCpG/ExpCpG ratio ranged from 1.35 to 1.50 in the Antarctic *pIgR* gene locus. Remarkably, no CGIs were found in the non-Antarctic relative *C. gobio*, nor in the temperate counterparts. The main features of the *T. bernacchii pIgR* gene locus described above are summarized in Figure 3.

### 2.2. Analysis of T. bernacchii pIgR cDNA

**Figure 4:**
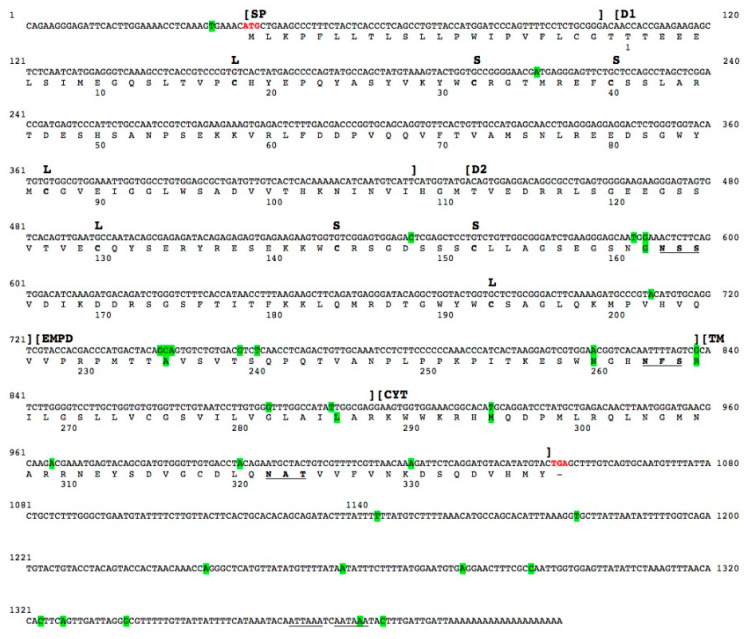
Nucleotide and deduced amino acid sequences of the gene encoding *T. bernacchii* pIgR. The start and stop codons are given in bold red, and the polyadenylation signals located downstream of the stop codon are underlined. The boundaries of the signal peptide (SP); the D1, D2, and EMPD domains; and the transmembrane (TM) and cytoplasmic (CYT) regions are indicated by square brackets above the nucleotide sequence. Polymorphic sites are highlighted in green. Amino acid residues are reported in one-letter code below the nucleotide sequence. The large (L) and the small (S) loops are indicated above codons for the cysteine residues forming them. N-glycosylation sites defined by the NXS/T sequons are bold and underlined. The numbering of nucleotides and of amino acids residues is reported above and below each sequence, respectively.

Initially, a partial cDNA sequence of the pIgR was obtained from total RNA extracted from the spleen of a *T. bernacchii* specimen, as described in the Section 4. The primers used for PCR experiments were designed on the nucleotide sequences encompassing the pIgR D1–D2 domains from *Epinephelus coioides*. The amplicon obtained (529 nt) was cloned and sequenced. Subsequently, to extend the 5′ end of cDNA, a 5′ RACE was performed by using the gene-specific primer pIGR1Rev, designed at the beginning of the D1 domain; the antisense primer pIGRIIr, designed in the middle of the D2 domain, was used for the nested PCR amplification. The amplicon obtained (235 nt) was cloned and sequenced. To complete the sequence at the 3′ end of the pIgR transcript, a 3′ RACE was carried out by using pIGR1Fwd as a sense-specific primer. To verify the correct amplification, a nested PCR reaction was performed, using pIGRII as a sense primer. The amplicon obtained (650 nt) was cloned and sequenced. The full-length cDNA sequence encoding *T. bernacchii* pIgR consisted of 1414 nt, including a 5′ UTR (38 nt) and a 3′ UTR (359 nt) (Figure 4). Two polyadenylation signals were identified, one canonical at position 1387, and a second one, non-canonical, at position 1395 (Figure 4). Thirty positions were polymorphic because differences were found in the transcript variants. Five carried non-synonymous substitutions. Interestingly, a fairly critical mutation was identified in the fourth position of the canonical polyadenylation signal, which blocks its function. In this circumstance, the non-canonical poly(A)-site is more likely to become functional.

The functional protein domains were identified in the deduced amino acid sequence using the bioinformatics tools SignalP, Prosite, and TMpred. Following a 21-aa long signal peptide, two immunoglobulin domains, D1 and D2 were found, consisting of 108 and 95 residues, respectively, separated by a short linker sequence (Figure 4). The D1 and D2 domains were both identified as IgV domains, since the two conserved cysteine residues forming the intrachain disulfide bond were found to be spaced by 68 aa (D1) or 62 aa (D2). This is a distance greater than that found in IgC- and IgI-type domains. Furthermore, the presence of two additional cysteine residues, spaced by seven amino acid residues in both domains, is a typical feature of the IgV domains of pIgRs. It was noted that, while D1 is encoded by a single exon, the D2 nucleotide sequence also comprises one intron (Figure S1), which is an unusual feature for IgV domains. A 39-aa extracellular proximal domain (EMPD) sequence, with a high theoretical pI (11.0), rich in prolines (17.9%), was preceding, at the carboxy-terminus of D2, the transmembrane domain (TM). The latter consisted of 20 residues and was characterized by a preponderance of leucine residues (30%) and the presence of a cysteine residue, very infrequent in transmembrane proteins (Figures S2 and S3). The sequence ended with a 51-aa-long basic cytoplasmic tail (Figure 4). The amino acid composition of *T. bernacchii* pIgR is reported in Table S2.

**Figure 5:**
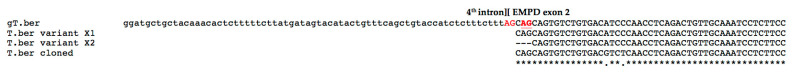
Alignment of the *T. bernacchii pIgR* genomic sequence with that of the three transcript variants. Of the *pIgR* genomic sequence (gT.ber), retrieved from the *T. bernacchii* genome, only the region spanning the 3′ end of the fourth intron and the 5′ end of the EMPD exon 2 is shown. Two pIgR variants (T.ber variant X1 and T.ber variant X2) are predicted from the genome; the *pIgR* cDNA variant (T.ber cloned) has been isolated in the present study. The canonical acceptor splicing site is depicted in red. The cryptic splicing site is in bold red and underlined. Gaps are indicated by dashes. Identical nucleotides are indicated with an asterisk below the alignment; positions differing in one nucleotide are marked by dots.

Once the cDNA sequence was obtained, it was aligned against the two *pIgR* transcript variants, X1 and X2, predicted from the reference genome (Figure 5).

The cloned sequence that resulted was identical to the variant X1, whereas the variant X2 had a deletion of a triplet that encodes an alanine in the 5′ end of the EMPD exon 2. Therefore, we evaluated putative alternative exon isoforms by applying a computational tool for the identification of potential splice sites. We highlighted that the site at position 73 in the region encompassing the 3′ end of the fourth intron and the 5′ end of the EMPD exon 2 showed the highest score as a cryptic-acceptor splicing site, thus accounting for the variant X2 (Table S3). 

**Figure 6:**
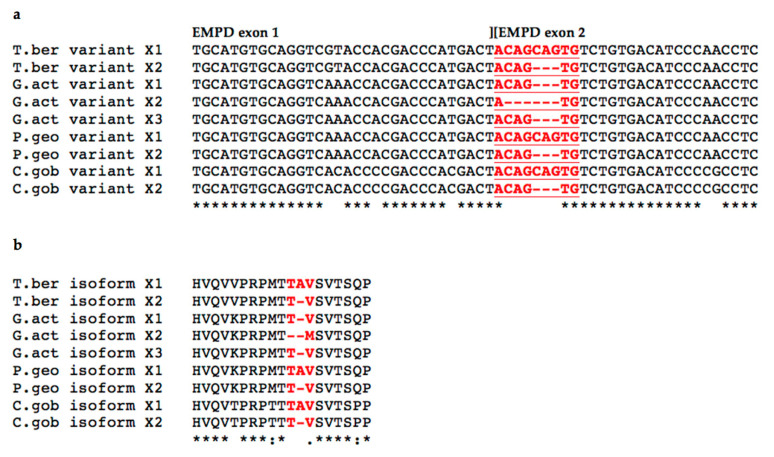
Multiple alignments of the EMPD sequences. (**a**) Multiple alignment of cDNA sequences spanning the two EMPD exons of the pIgR variants identified in the Antarctic species *T. bernacchii* (T.ber variants X1 and X2), *G. acuticeps* (G.act variants X1, X2, and X3), *P. georgianus* (P.geo variants X1 and X2), and in the non-Antarctic species *C. gobio* (C.gob variants X1 and X2). The exonic region in which a cryptic splicing site was predicted is depicted in bold red and underlined; (**b**) multiple alignment of deduced amino acid residues of the EMPD domain. Amino acid residues involved in the usage of the cryptic splice site are depicted in bold red. Gaps are indicated by dashes. Identical nucleotides or amino acid residues are marked with an asterisk below alignments; positions differing in one nucleotide or amino acid residue are marked with a dot.

To further extend the analysis of the *pIgR* gene structure in cold-adapted species, two more Antarctic species, *G. acuticeps* and *P. georgianus*, were added along with the non-Antarctic species *C. gobio*, since complete reference genomes were available for all of them. Two (in *P. georgianus*) or three (in *G. acuticeps*) *pIgR* transcripts, referred to as variant X1, variant X2 and variant X3, were annotated in the respective genome assemblies (Figure 6). 

**Figure 7:**
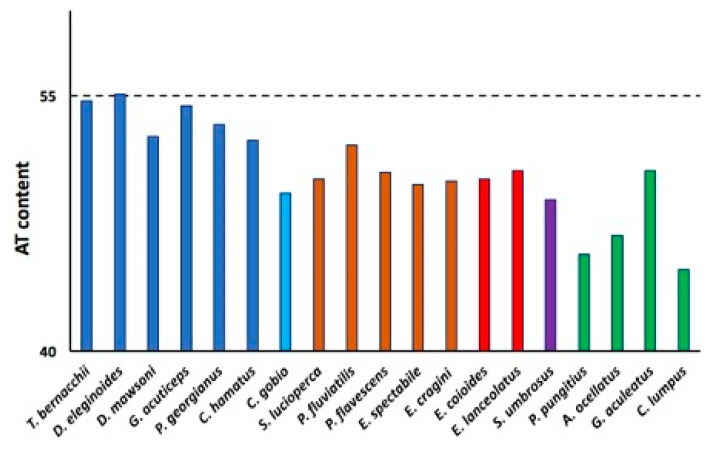
Percentage of AT content of the *pIgR* exons of Antarctic species (blue bars) and the non-Antarctic notothenioid species *C. gobio* (light blue bar) compared to representative species of the temperate perciform suborders Percoidei (brown bars), Serranoidei (red bars), Scorpaenoidei (purple bar), and Cottoidei (green bars).

As previously shown by the AT content analysis of notothenioid *IgT* genes [21], a remarkably high AT content of *pIgR* exons was shared within Antarctic species, reaching a peak (55.8%) in the Nototheniidae family, which comprises *T. bernacchii* and *Dissostichus eleginoides* (Figure 7). This finding reflects a peculiar feature not shared with either the non-Antarctic species *C. gobio*, their closest relative, or all the other Perciformes.

### 2.3. Analysis of T. bernacchii pIgR-Deduced Amino Acid Sequence

To identify the adaptive characteristics unique to notothenioid pIgR sequences, we extended our analysis to other notothenioid species and representatives of the five perciform suborders on the basis of the available data (see Section 4.1). However, since many sequences were fragmentary and/or incomplete, for convenience, the overall comparisons only referred to the overlapping regions. 

**Figure 8:**
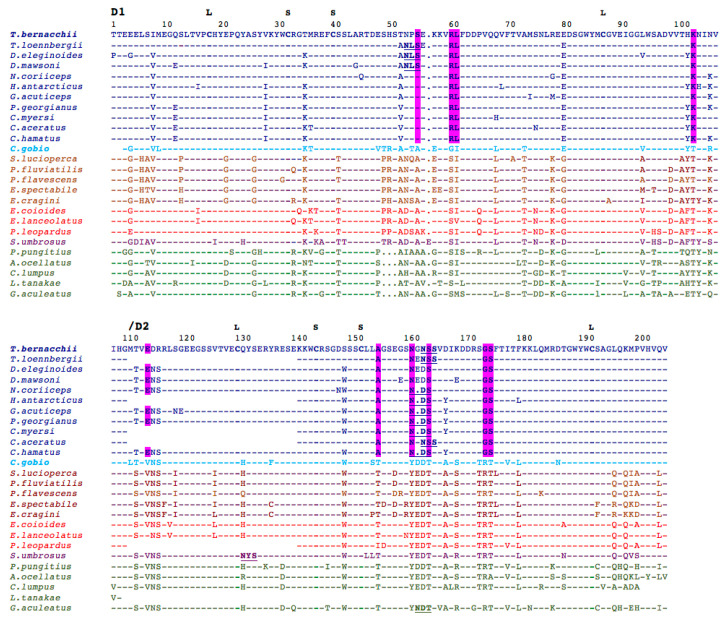
Multiple amino acid sequence alignments of pIgR D1–D2 domains from Notothenioidei (in blue; *T. bernacchii* in bold blue, and *C. gobio* in bold light blue) and other species of the perciform suborders Percoidei (in brown), Serranoidei (in red), Scorpaenoidei (in purple), and Cottoidei (in green). Antarctic-notothenioid-specific residues are reported in bold and highlighted in magenta. The highly conserved cysteine residues, characteristic of the immunoglobulin fold, are in bold. The large (L) and the small (S) loops are indicated above the cysteine residues forming them. Putative N-glycosylation sites are in bold and underlined. Amino acid residues that are identical to those shown in the sequence of *T. bernacchii* are indicated by dashes. Gaps are indicated by dots. The complete alignment is shown in Figure S3.

Some conserved motifs, previously identified in other fish and suggested to help stabilize the secondary structure of pIgR [14], were also found in D1 (CWDC, KYWC, and DxGxYxC motifs) and D2 of *T. bernacchii* pIgR (KxWC and DxGWYWC) (Figure 8).

Twenty amino acid positions were found to be specific for Antarctic pIgRs, as present in all Antarctic sequences but absent in all sequences from the other species analyzed, as well as in the non-Antarctic notothenioid *C. gobio* (Figure S3). Ten were present in the secretory component; four positions (S55, R60, L61, and K103) were localized in the D1 domain, six in D2 (E114, A155, N161, S164, G174, and S175) (Figure 8), one in the EMPD, three in the TM domain, and six in the cytoplasmic tail (Figure S3). In addition, it is noteworthy that five out of the 10 notothenioid-specific residues, which are present in the extracellular portion of the receptor, introduce or abolish an electrostatic charge; N161 and S164 are convergent substitutions since both introducing a glycosylation sequon NXS/T (where X is different from P). Two additional residues (E80 and T112) were shared by the Antarctic and non-Antarctic nothotenioid species, but they were absent in the other perciform suborders (Figure 8).

**Figure 9:**
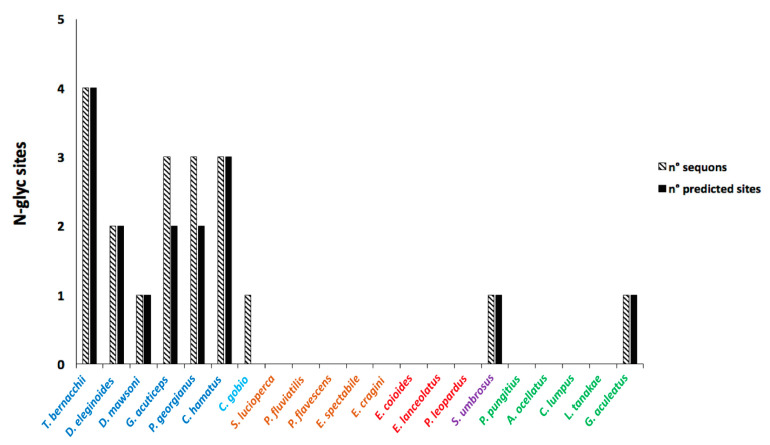
Distribution of potential N-glycosylation sequons (diagonal black bars) and glycosylated sequons (black bars) in the pIgR of Antarctic (blue) and non-Antarctic (light blue) notothenioid species compared to representative species of the temperate perciform suborder Percoidei (brown), Serranoidei (red), Scorpaenoidei (purple), and Cottoidei (green).

Glycosylation was found to be another distinctive feature of the notothenioid pIgR. Up to four N-glycosylation sequons were found in Antarctic fish compared to the other perciform species, which showed no sequons at all, except for *Sebastes umbrosus* and *G. aculeatus*, harboring just one (Figure 9). Interestingly, the site in D2, which is present in 9 out of 12 sequences, is alternately located at two asparagines spaced by a single residue (Figure 8).

**Figure 10:**
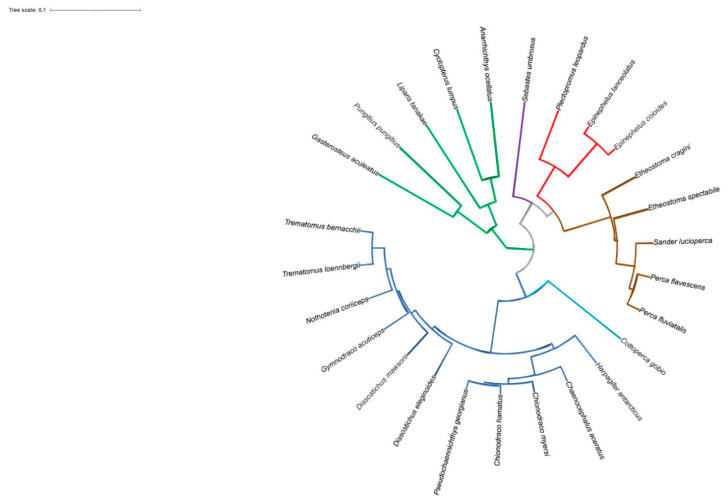
Distance tree of the pIgR D1 domain from teleost species belonging to the perciform suborders Notothenioidei (Antarctic species, blue lines; non-Antarctic species, light blue line), Percoidei (brown lines), Serranoidei (red lines), Scorpaenoidei (purple lines), and Cottoidei (green lines). The tree was generated by the Clustal Omega tool. The sequences used are in Figure 8.

Interestingly, all the sequons identified were predicted to be glycosylated in most Antarctic species, except for two out of three in *G. acuticeps* and *P. georgianus*. Of the four notothenioid N-glycosylation sites, one occurs in each of the D1 and D2 domains, one at the boundary between the TM region and the extracellular portion, and another in the cytoplasmic tail (Figure S3). The distance tree generated from the multiple alignments clearly confirms phylogenetic relationships among the species analyzed (Figure 10).

### 2.4. Structural Analysis of T. bernacchii pIgR

**Figure 11:**
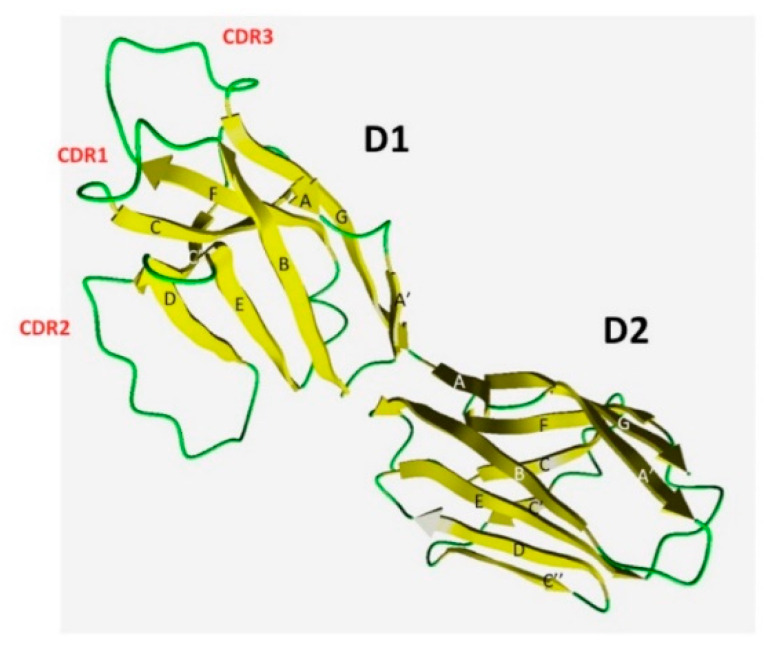
Ribbon representation of the molecular model of *T. bernacchii* pIgR extracellular region. The β-strands are labeled with uppercase letters (A–G). Loops that correspond to the three Complementary Determining Regions in the D1 domain are labeled (CDR) and colored (green).

We constructed a molecular model of the ectodomain of *T. bernacchii* pIgR. It consists of two tandem IgV domains, D1 and D2, whose axes diverge at about 120°. Differently from other IgV domains, a disulfide bridge connects C and C’ strands in both D1 and D2 domains. The regions that are defined as complementary determining regions (CDRs) in the antibody VH and VL domains were recognized in the D1 domain (Figure 11). D1 CDR loops are more extended, while the structure of D2 appears more compact and contains an additional C’’ strand. Minor structural differences between trout, selected as the template structure, and *T. bernacchii* are related to the size of D1 CDR2, which is longer in *T. bernacchii* (Figure 11).

The N-glycosylation sites of D2 were exposed to solvent (Figure S4), suggesting a role of the attached carbohydrate moiety. Notably, all substitutions introducing electrostatic charges in notothenioids were also found to expose the side chain to the solvent as well as the glycosylated N161, suggesting that the solubility of the molecule increases at the very low temperature of Antarctic seawater. The position of the Antarctic species-specific charged residues R60, K103, E114, and N161 is shown in Figure S4.

### 2.5. Basal Expression Analysis of pIgR Transcripts in T. bernacchii Mucosal Tissues and Lymphoid Organs 

**Figure 12:**
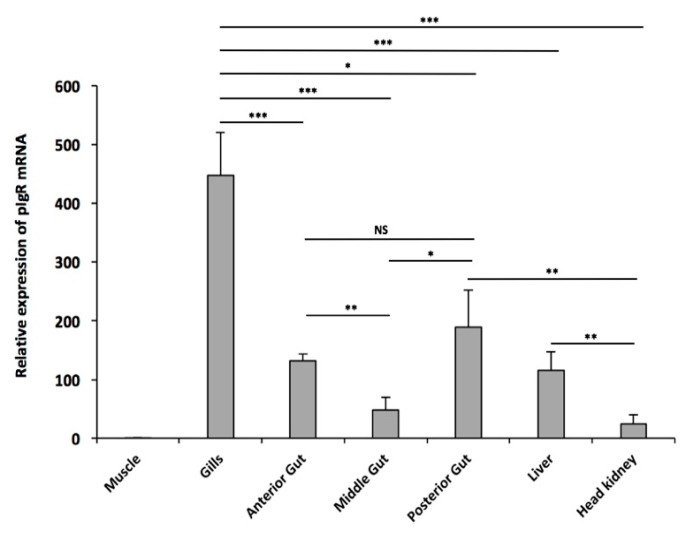
Relative expression levels of *pIgR* in different tissues from *T. bernacchii*. Data from three independent experiments are presented as mean gene expression relative to the housekeeping β-actin (±SD). The muscle tissue was used as a negative control. Levels of transcription were evaluated by q-PCR in duplicates using three *T. bernacchii* specimens. * *p* < 0.05; ** *p* <0.01; *** *p* < 0.001; NS, not significant (two-tailed Student’s *t* test with Bonferroni correction).

To gain some insights into the constitutive expression of *pIgR* in a cold-adapted teleost, a relative mRNA expression pattern of the gene was determined by q-PCR in mucosal tissues and lymphoid organs of *T. bernacchii*. As shown in Figure 12, *pIgR* transcripts were expressed in all the tested tissues, with the highest abundance detected in the gills and the lowest in muscle, as expected. Higher levels were also found in the intestinal segments. In particular, the mRNA levels in the middle intestine were 2.7-fold (adjusted *p* < 0.01) and 3.9-fold (adjusted *p* < 0.05) lower than those in the anterior and posterior ones, respectively. No statistically significant transcriptional differences were found between the anterior and posterior segments. A moderate *pIgR* expression was detected in the liver and head kidney, the former being 3.9-fold and the latter 10-fold lower than gills (adjusted *p* < 0.001). These findings are consistent with the predominant role of pIgR in mucosal compartments.

### 2.6. pIgR Expressing Cells in T. bernacchii Intestinal and Hepatic Tissues

**Figure 13:**
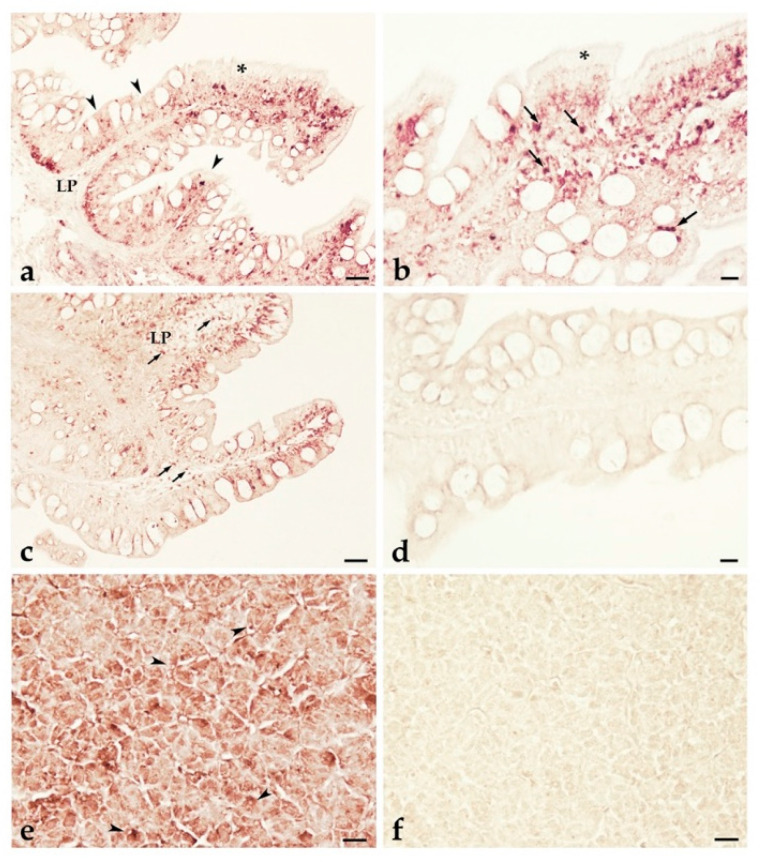
ISH of *T. bernacchii pIgR*. *pIgR*-expressing cells detected in *T. bernacchii* (posterior) gut (**a**–**c**) and liver (**e**) using an antisense probe. No signals were detected in either intestine (**d**) or liver with a sense probe (**f**). Stained enterocytes and liver epithelial cells are shown by arrowheads. Scattered cells containing *pIgR* transcripts are mainly in the epithelium and in the lamina propria (arrows). LP, lamina propria; * apical surface of enterocytes. Scale bars: 20 μm.

To identify pIgR-producing cells in *T. bernacchii* tissues, we performed ISH analysis with anti-sense and sense RNA DIG-labeled probes. Given the results obtained by q-PCR, we paid special attention to the gut–liver communication axis. The posterior intestine, which displayed higher *pIgR* expression levels (Figure 12), was lined by a simple columnar epithelium of polarized cells (enterocytes) (Figure 13a–d). All the enterocytes were *pIgR*-expressing cells (Figure 13a–c). In particular, the highest staining intensity was detected around the nucleus and on the enterocyte basolateral surface, which faces the basement membrane (Figure 13b). The apical surface of most epithelial cells did not display any staining. Scattered cells, localized both in the epithelium (Figure 13b) and the underlying lamina propria, were stained (Figure 13c). Moreover, the staining with the anti-sense probe (Figure 13e) revealed a strong signal throughout the liver. Notably, the expression of the *pIgR* gene was mainly detected around the nucleus of most hepatocytes. ISH with the pIgR sense probe did not result in any staining both in the posterior intestine and liver, as expected (Figure 13d–f).

## 3. Discussion

The pIgR plays a crucial role in mammalian immune responses since it ensures multifaceted immune functions [14,23]. The pIgR has been studied in multiple teleost species, highlighting similarities and differences in its structure, as reviewed by Kortum et al. [15]. However, data about teleost pIgR functions are very limited and mainly refer to the transport of pIgs across the mucosal epithelial cells [2,11,13,14,15]. At present, the *pIgR* gene locus organization still remains poorly investigated. The *pIgR* gene has been identified as a single copy in zebrafish, along with a wide multigene family that comprises *pIgR*-*like* (pIgRL) genes, differentially expressed in lymphoid and myeloid cells [15]. A BLAST search of the *Takifugu rubripres* genome database allowed identification of a *pIgR* homologous gene [24].

To expand the current knowledge in this field, we isolated and characterized, for the first time, the *pIgR* gene in the cold-adapted species *T. bernacchii*. Thanks to the increasing number of sequenced genomes from teleosts [25] over the past few years, we searched the annotated genome of *T. bernacchii* [26] to perform a full analysis of the *pIgR* gene structure. Additionally, the genome assemblies of two other cold-adapted species *G. acuticeps* and *P. georgianus* [26], and *C. gobio* [27], a temperate notothenioid species phyletically basal for the Antarctic Clade [18], were searched for a sequence-based comparison. We identified a single *pIgR* gene composed of eight exons and seven introns, as reported for other fish *pIgRs*. Notably, we found that Antarctic species have the largest *pIgR* gene (8235–9013 nt) compared to the other Perciformes included in the present study (3700–7090 nt). *C. gobio* showed a 5930-nt long gene, which was shorter than the Antarctic species but closer to the size of the *pIgR* gene from the temperate species. Further investigation found that the main difference with the temperate orthologs was in the length of introns, e.g., the third and fourth introns were twice or three times longer. The second intron was particularly long in the Antarctic species, while the third intron in *C. gobio* showed a standard length, as found in the temperate perciforms. These findings faithfully mirror the phylogenetic relationships among Notothenoidei [28], as also confirmed by the distance tree of the pIgR D1 domain from notothenioid fish and species belonging to the other perciform suborders. 

The expression of the *pIgR* gene still needs further analysis, as a single transcript of this gene is usually found in most fishes. However, in *Ctenopharyngodon idella*, seven *pIgR* splicing transcripts were identified, a full-length and six truncated variants, two generated by exon skipping, and the other four having different motif arrangements at the 3′ end [29]. In the present study, we determined that the introns of the *pIgR* gene lengthened during the evolution of Notothenioidei without impairing the splicing process, as confirmed by the identification of transcript variants relative to the 5′ EMPD exon 2. Using a splice prediction tool, we examined several sequence features of constitutive and cryptic sites and identified alternative isoforms. Alternative splice sites are generally defined as weaker than the constitutive ones [30]; however, in the case of the *T. bernacchii pIgR* gene, the score relative to the variant X2, which derived from the usage of a cryptic site, was almost as high as that of the constitutive acceptor. The presence of the variant X2 remains to be assessed, as it is only predicted by genome evaluation. Conversely, the genomic variant X1 was readily aligned to the cDNA sequence.

Intron size can vary due to the accumulation over time of non-homologous recombination, insertions and deletions, and TE activity. TEs account for a significant portion of vertebrate genomes and are known to play a role in genome modifications [31,32]. Hotspots of retrotransposons (Rex-like) and DNA transposons (Tc1-like) elements have been reported in multiple teleost fishes, including Notothenioidei [26,33,34]. These findings support the idea that TEs could have contributed to the evolutionary process that led to the elongation of introns observed in Antarctic *pIgR* genes. The search for TEs in the introns of nototheniods and of the other five perciform suborders confirmed the presence of several elements. Notothenioidei contain all types of TEs, whereas some temperate species possess just one type, e.g., DNA transposons in *S. umbrosus* (Scorpanoidei) and *A. ocellatus* (Cottoidei) or LINEs in *P. fluvialitis* and *P. flavescens* (Percoidei). The prevalence of DNA transposons in the temperate species, including the non-Antarctic species *C. gobio*, along with the absence of TEs in multiple species belonging to different suborders, may be related to the evolutionary modifications of the whole teleost genome [35]. It is notable that the retrotransposons became prevalent in Antarctic fish. A detailed analysis of the distribution of TE elements in each intron interestingly underlined that in *T. bernacchii*, the first and second introns were characterized by the presence of one type of TEs, while the third intron appeared to be more heterogeneous due to the presence of SINEs and LINEs. More interestingly, SINE elements found in the third intron of *T. bernacchii* represented the highest percentage (7.3%) of TEs identified in the other cold-adapted species. It is well known that SINEs are frequently found in Trematominae genera, accounting for their rapid radiation/genome rearrangement [36,37]. To our knowledge, this finding is not surprising. Previous studies we conducted on the Ig heavy chain gene locus of Notothenioidei revealed peculiar rearrangements of the intronic regions of both *IgT* and *IgM* heavy chain genes [21,38]. 

In light of these data, we can hypothesize that in cold-adapted species, the long intronic sequences, other than the first ones, may have evolved under selective constraint since they are functionally relevant. The primary role of some introns in regulating gene expression has been described well over the past decades [39]. Prediction of the binding sites for NF-κB, STAT6, and IRF1 in the sequences of fish *pIgR* genes available in GenBank allowed their consideration as potential regulators in fish [14], in line with the cytokine-inducible regulatory elements, which are well-known to direct the transcription of *pIgR* in mammals [13,40]. Our analysis clearly evidenced that the second and third introns in Antarctic species were enriched for transcriptional factors, different from the other introns and from the other species analyzed. Given the structure of the *pIgR* gene, which comprises more exons with multiple variants, the presence of intragenic transcription factors identified in Antarctic teleosts may suggest a fine-tuned regulation of its expression. At present, the regulatory mechanism of *pIgR* expression has not yet been clarified in teleost fish nor in cold-adapted species. Some insights into the adaptive strategies employed to ensure efficient gene expression under cold conditions were described by Lau et al. [41]. These authors proposed that Antarctic fishes may have adjusted the transcription of their globin genes by duplicating *cis*-acting regulatory elements. 

Another clue for the possible fine-tuning of the gene regulation of *T. benacchii pIgR* was provided by the presence of CpG islands (CGIs). It is well-known that in vertebrate genomes, CGIs are involved in transcriptional regulation [42]. The identification of promoter-associated CGIs has interesting implications for a possible key role of epigenetic regulation in cold-adapted *pIgR* genes. In addition, Varriale and Bernardi [43] reported an increase in CGIs and DNA methylation levels in Antarctic fishes, most likely accompanied by the progressive cooling of Antarctic seawaters. However, the nature of regulatory elements and the mechanisms underlying transcription at low temperatures are still poorly known and deserve further study. It is tempting to experimentally verify the *pIgR* gene regulation in cold-adapted teleosts. Further, assessing whether CGI sites are differentially methylated in a tissue-specific manner might help evaluate DNA methylation patterns in response to environmental threats.

In contrast to human pIgRs, which are extensively glycosylated, fish pIgRs show limited or even no glycosylation sites. These data raise the question of whether N-glycosylation of pIgR is necessary for its function. This is an interesting issue that became even more relevant when we highlighted the presence of up to four N-glycosylation sites exclusively in pIgRs from Antarctic species. In addition, all sites were predicted to be glycosylated, thus appearing as a distinctive feature of coldwater species. In particular, the asparagine residue of the glycosylation sequon present in the extracellular portion of *T. bernacchii* pIgR was found to be exposed to solvent in the molecular model we built, indicating the carbohydrate availability for binding. Generally, carbohydrates are involved in protein folding, stability, and protection from proteolytic attacks. Glycosylation can also modulate a correct balance between protein solubility and structural flexibility. This role can be viewed as a special adaptive response to a cold environment, such as in the case of Antarctic fish IgT and IgM, which were found to be highly glycosylated [21,44]. A further element that indicates an increase in solubility is related to the greater surface electrostatic charge. In fact, some amino acid residues, specific to the cold-adapted species, introduced electrostatic charges located on the surface of our molecular model. These findings underline several peculiar features that may be considered hallmarks of cold pIgRs.

Furthermore, *pIgR* expression was analyzed in mucosal tissues and lymphoid organs, including the anterior, middle, and posterior intestine; gills; and head kidney. *T. bernacchii pIgR* transcripts were predominately expressed in the mucosal tissues, showing similar expression patterns to those of other teleosts species [10,15,24,45,46,47]. The highest *pIgR* level detected in the gills is one of the most interesting aspects. Considering that the gills represent one of the first lines of defense in teleosts, it will be challenging to assess whether the *pIgR* gene expression is upregulated in a tissue-specific manner in *T. bernachii*. Studies on CGIs and/or DNA methylation can provide additional insight into the regulation of the *pIgR* in response to pathogens, which are naturally occurring in the cold-adapted fish investigated, e.g., the well-documented nematode parasite infection [48,49]. In other teleost species, high *pIgR* expression was found in gills after bacterial infection was induced [11,14]. Additionally, *pIgR* mRNA had higher expression in the posterior intestine than in the middle segment and did not differ from the anterior segment. These results, in line with the findings collected from other teleost fish [45,50], further confirmed that the posterior intestine plays an important role in the mucosal immune response and host defense. 

High staining intensity was found through ISH in the cytoplasm of the enterocytes, suggesting that *T. bernacchii* pIgR mediates transepithelial transcytosis, as described in mammals [23]. Scattered *pIgR*-expressing cells were also detected in the mucosa, as previously observed in *Cyprinus carpio* [45]. However, currently, there are not yet any markers to provide evidence of the nature of these *pIgR*-expressing cells, although staining may suggest an additional role played by pIgR molecules in teleost fish. In mammals, pIgR ensures multifaceted immune functions, such as (i) improved pIg stability, (ii) protection of secretory Ig from proteolytic degradation, (iii) the exclusion of pathogens from mucosal surface, (iv) the intracellular neutralization of invading pathogens, (v) the removal of pathogen-secretory Ig complexes from infected tissues, and (vi) the nonspecific microbial scavenger function of the free secretory component (SC) [23]. Data about these functions of pIgR in teleost mucosal immunity are very limited, although new findings provided direct evidence for pIgR-mediated immune excretion of IgM–antigen complexes in *Paralichthys olivaceus* [51].

The role of the liver was considered to expand the current knowledge about the *pIgR* expression and pIgR-mediated transport of Sigs in teleost hepatocytes [22,52,53]. The results obtained by q-PCR demonstrated that *pIgR* was expressed in *T. bernacchii* liver, and ISH demonstrated that the transcripts were localized around the nucleus of the hepatocytes. In this species, IgM-immunoreactivity was detected in the perisinusoidal cells, bile canaliculi, pre-ductules, and the intraluminal mucus of the anterior intestine [22]. The data strongly substantiate that *T. bernacchii* IgM may be transported via pIgR across the hepatocytes to be secreted into the bile and, subsequently, into the gut. This implies the putative formation of a receptor-Ig complex, as is the case in mammals [52,53]. Numerous observations indicated that teleost pIgR binds IgM [24,47,54] and IgT [55,56], while it remains to be elucidated whether this can occur for IgD. We consider that to improve the current understanding of the *T. bernacchii* pIgR/SIg system and reveal the specific mechanisms of intracellular Ig transport, it will be necessary to rely on the availability of specific markers and adopt novel technologies from the field of molecular cell biology. Addressing these and other future studies will aid in further dissecting the complex roles of pIgR in the mucosal immune defense of teleost fish. 

In conclusion, these findings highlight several peculiar features acquired by cold-adapted pIgR and underpin its pivotal role played in mucosal immune defense. The genome information gained in the present study will be useful for comparative and functional genomic analyses and contribute to advancing the current knowledge of the *pIgR* gene in teleost fish.

## 4. Materials and Methods

### 4.1. Biological Samples

Adult female specimens of *Trematomus bernacchii* (Nototheniidae family) were caught by use of nets in the Ross Sea, in the proximity of the Italian “Mario Zucchelli” Station at 74°42′ S, 164°07′ E during the XXV Italian Antarctic Expedition (2009–2010). The activity permit, released by Italian National Program for Antarctic Research (PNRA), was in agreement with the “Protocol on environmental protection to the Antarctic Treaty” Annex V. Fish specimens (average weight 350 g) were kept in aquaria with running, aerated seawater until sacrificed. Tissues were collected and immediately frozen in liquid nitrogen.

### 4.2. Cloning of pIgR Transcript

Total RNA was extracted using an SV Total RNA Isolation System kit (Promega, Madison, WI, USA) from 150 mg of head kidney collected from a *T. bernacchii* specimen, homogenized by Potter-Elvehjem glass–Teflon. RNA quality was assessed on a 2% agarose gel and by measuring A260/A280 ratio; the concentration was assessed by reading absorbance at 260 nm with a NanoDrop 1000 Spectrophotometer (Thermo Fisher Scientific, Waltham, MA, USA). RNA was then subject to DNase I treatment (Thermo Scientific, #EN0521) in order to avoid DNA genomic contamination for downstream analysis. cDNA was obtained from 1 μg of total RNA using Maxima H Minus Reverse Transcriptase (Thermo Scientific, #EP0751). The oligonucleotides used as primers to perform the first round-PCR amplification were designed on the nucleotide sequence coding for the D1–D2 domains of *E. coioides* pIgR (accession number FJ803367), available in the GenBank database. The target sequence was amplified in a final volume of 25 μL using 2 μL cDNA (20 ng), 1.25 μM of specific primers (1.0 μM), 0.5 μL of dNTP Mix (0.2 μM), 2.5 μL 10X DreamTaq Buffer, and 0.5 μL (1 U) of DreamTaq DNA polymerase (Thermo Scientific, #EP0705), up to volume with H_2_O as follows: 95 °C for 3 min, 35 cycles of 95 °C (30 s), 60 °C (30 s), and 72 °C (1 min), with a final extension at 72 °C for 10 min. In order to improve the yield of the specific target amplification, the PCR product was then subject to a second amplification, following the same conditions as the primary PCR. Primers used in all the PCR experiments are shown in Table S4, which also reports the target domains (D1–D2) of pIgR for each primer. PCR products were analyzed on a 1.5% agarose gel, subsequently purified by NucleoSpin^®^ Gel and PCR Clean-up (Macherey-Nagel, Düren, Germany), and finally cloned into pGEM^®^-T Easy Vector (Promega, #A1360). Positive clones were screened by the blue/white method and sequenced on both strands on an ABI PRISM 3100 automated sequencer at Eurofins Genomics Europe Sequencing GmbH (Jakob-Stadler-Platz 7, 78467 Konstanz, Germany).

### 4.3. 3′ and 5′ Rapid Amplification of cDNA Ends (RACE)

In order to complete the 3′ cDNA region of the *T. bernacchii pIgR*, 3′ Rapid Amplification of cDNA Ends (3′RACE) was performed using a commercial kit (Invitrogen by Thermo Fisher Scientific, Waltham, MA, USA) according to the manufacturer’s instructions. First-strand cDNA was synthesized as described above, using AP as a specific primer. PCR amplification was then carried out with pIGR1Fw as a sense primer and AUAP as an antisense primer. Subsequently, a nested PCR was performed with pIGRII as a sense primer and AUAP as antisense primer (Table S4). The amplification was performed as follows: 95 °C for 5 min, 40 cycles of 95 °C (30 s), 55 °C (30 s), and 72 °C (1 min) with a final extension at 72 °C for 15 min. Then, 5′ Rapid Amplification of cDNA Ends (5′ RACE) was carried out on *T. bernacchii pIgR* cDNA using 5′ RACE System for Rapid Amplification of cDNA Ends version 2.0 (Invitrogen™), following the manufacturer’s instructions. First-stranded cDNA was synthesized using an antisense-specific primer pIGR1Rev. Subsequent PCR amplification was performed with pIGR1Rev and Oligo d(T)-anchor primer (AAP), supplied by the kit as sense primer. A nested PCR was performed with pIGRIIr and AAP. The amplification was performed as follows: 95 °C for 3 min, 40 cycles of 95 °C (30 s), 60 °C (30 s), 72 °C (1.30 min), with a final extension at 72 °C for 10 min. Then, 3′ and 5′ RACE products were cloned and sequenced as described above.

### 4.4. Data Availability

The cDNA sequence coding for pIgR from *T. bernacchii* has been deposited in the GenBank database (https://www.ncbi.nlm.nih.gov/genbank/) on 8 July 2021 under the accession number MZ540772. The nucleotide sequences from the other species used for molecular analysis are reported in Table S5. Genome assemblies and predicted transcripts of pIgR from the Antarctic species *T. bernacchii* (v. fTreBer1.1), *G. acuticeps* (v. fGymAcu1.1), and *P. georgianus* (v. fPseGeo1.1) [26]—for all species above from the temperate notothenioid species *C. gobio* (v. fCotGob3.1) [27]. The scorpaenoid *S. umbrosus* genome assembly (v. fSebUmb1.pri) is within the framework of the Vertebrate Genome Project (https://www.ncbi.nlm.nih.gov/bioproject/PRJNA562006/, accessed on October 2021) [25].

*pIgR* genome assemblies and predicted transcripts from *S. lucioperca* (v. SLUC_FBN_1.2) [57], *P. fluviatilis* (v. GENO_Pfluv_1.0) [58], *P. flavescens* (v. PFLA_1.0) [59], *E. spectabile* (v. UIUC_Espe_1.0) [60], *E. cragini* (v. CSU_Ecrag_1.0) [61], *E. lanceolatus* (v. ASM528154v1) [62], *P. leopardus* (v. YSFRI_Pleo_2.0) [63], *P. pungitius* (v. NSP_V7) [64], *A. ocellatus* (v. GSC_Weel_1.0) [65], and *G. aculeatus* (v. GAculeatus_UGA_version5) [66] were retrieved from research articles that first reported them. 

*pIgR* transcripts from the Antarctic species *D. eleginoides* [67], *N. coriiceps* [68], and *C. hamatus* [69], and from the serranoid species *E. coioides* [10], were retrieved from transcriptome shotgun assemblies, whereas for the Antarctic species *D. mawsoni* [70], *H. antarcticus* [26], *C. myersi* [71], and *C. aceratus* [72], *pIgR* transcripts were retrieved from genome assemblies.

### 4.5. Gene Sequence Analyses

The *pIgR* intronic sequence dataset derived from all the species considered was scanned by using different tools. The MEME (Multiple Em for Motif Elicitation) tool within the MEME suite v.5.4.1 environment [73] was used to discover conserved motifs by using default parameters. In order to verify whether introns harbor repeated elements, all sequences were analyzed with the RepeatMasker software v.4.09 [74], which is based on the annotations available at Ensembl, and we screened the repeats against the Dfam v.3.5 [75] library of known repeats found in the genomes of Actinopterygii, excluding simple repeats or low-complexity DNA. All introns were queried for the presence of transcription factor binding sites using the online tool Tfsitescan (www.ifti.org/cgi-bin/ifti/Tfsitescan.pl) [76]. A sequencing chromatogram was visualized using the program FinchTV (version 1.3.0). The nucleotide sequence obtained was verified by sequence similarity searches against the GenBank database using the BLAST program. The prediction of cryptic and constitutive splice sites in *pIgR* from *T. bernacchii* was performed by using the Alternative Splice Site Predictor (ASSP) Tool [77]. Polyadenylation signals in the *pIgR* nucleotide sequence were predicted using Poly(A) Signal Miner [78]. The AT content of cDNA sequences was calculated with the GC Content Calculator (Biologics International Corp, Indianapolis, IN, USA).

### 4.6. Deduced Amino Acid Sequence Analyses

The amino acid sequences were deduced from nucleotide sequences using the ExPASy Translate Tool (https://web.expasy.org/translate/). The amino acid composition was analyzed using the ProtParam [79] and Pep-Calc (www.pepcalc.com) tools. The transmembrane-spanning regions and their orientation were predicted by using the TMpred tool [80]. The presence of the signal peptide was determined by using the SignalP-6.0 Sever tool [81]. Multiple sequence alignments were performed with Clustal Omega (https://www.ebi.ac.uk/Tools/msa/clustalo/) [82], and the out alignments were obtained in ClustalW format [83]. A distance-based tree of the D1 sequences Clustal Omega out alignment was reconstructed and analyzed by the iTOL program (https://itol.embl.de/) [84]. Sequons and putative N-glycosylation sites were identified using the NetNGlyc 4.0 Server [85]. A 3D molecular model was built for the *T. bernacchii* pIgR ectodomain using the Phyre2 tool (http://www.sbg.bio.ic.ac.uk/phyre/html/) [86]. A total of 90% of the amino acid residues were modeled at 100% confidence using the 5f1s PDBe template (*Oncorhyncus mykiss* pIgR). A Phyre molecular model was also built for the transmembrane domain. The highest confidence was 69% with the 6rx4 PDBe template. The obtained PDB models were analyzed by the molecular graphics program YASARA [87] (www.yasara.org).

### 4.7. Expression Analysis of pIgR Using Real-Time PCR

Total RNA was extracted using an SV Total RNA Isolation System kit (Promega) from 150 mg each of anterior, middle, and posterior gut, liver, gills, head kidney, and muscle collected from three *T. bernacchii* specimens. Quantitative PCR-based expression analysis was performed on *T. bernacchii* cDNA using the Light Cycler 480 (Roche, Basel, Switzerland). The reaction consisted of 2 μL of cDNA diluted 1:10 and mixed with 5 μL of PowerUp™ SYBR™ Green Master Mix 2X (Applied Byosystem™) in a final volume of 10 μL with a final concentration of 0.3 μM of each primer, according to the manufacturer’s instructions. TbrtpIgRFwd and TbrtpIgRRev are primers designed on the D1 and D2 domains, respectively, for the amplification of products from *T. bernacchii* pIgR (Table S4). qPCR was performed three times, and samples, including DEPC water as a negative control, were run in duplicate each time. The PCR amplification conditions were 95 °C for 2 min, followed by 40 cycles of 95 °C (15 s), 60 °C (15 s), and 72 °C (1 m). In order to assess the amplification specificity and the absence of primer dimers, a final dissociation step was run to generate a melting curve. In all melting curve analyses, single specific peaks were observed. The relative expression of *pIgR* was determined with the 2^−ΔΔCq^ method, using β-actin as the housekeeper gene (Table S4) and muscle as the negative control.

Comparison between mucosal and lymphoid tissues was performed using two-tailed paired Student’s *t*-tests adjusted by Bonferroni post hoc test. Data are presented as means ± standard deviation. *p* values < 0.05 are considered as statistically significant, and shown as * *p* < 0.05, ** *p* < 0.01, and *** *p* < 0.001.

### 4.8. In Situ Hybridization (ISH)

#### 4.8.1. Synthesis of RNA Probes

Cells from gills of *T. bernacchii* were obtained by tissue teasing and suspended in Tripure (Roche). Total RNA was isolated and resuspended in DEPC-treated water. For reverse transcription, the BioScript RNase H minus (Bioline) enzyme was employed, using 1 μg of total RNA and 0.5 μg of random primers [pd(N)6]. Specific PCR primers (Table S6) were designed to amplify a 419 nt product corresponding to the *T. bernacchii* pIgR sequence.

Reactions were carried out in an Eppendorf Mastercycler personal (Milano, Italy). The cycling conditions were 1 cycle of 94 °C for 5 min, 35 cycles of 94 °C for 45 s, 52 °C for 45 s, 72 °C for 45 s, followed by 1 cycle of 72 °C for 10 min. The resulting DNA was purified using the QIAquick Gel Extraction Kit (QIAgen) inserted into the pGEM-T Easy vector (Promega) and transfected into competent JM109 *E. coli* cells. Plasmid DNA from three independent clones was purified using the Wizard Plus SV Minipreps DNA Purification System (Promega) and sequenced using Eurofins Genomic Sequencing Services. Sequence similarity searching was carried out using the BLAST program. Selected plasmid clones were used as a target in PCR reactions to synthesize the anti-sense and sense probes (see primers in Table S6). PCR conditions for the anti-sense probe were 1 cycle of 94 °C for 5 min, 35 cycles of 94 °C for 45 s, 54 °C for 45 s, 72 °C for 45 s, followed by 1 cycle of 72 °C for 10 min. The cycling protocol used for the sense probe (the negative control of ISH experiments) was 1 cycle of 94 °C for 5 min, 35 cycles of 94 °C for 45 s, 48 °C for 45 s, 72 °C for 45 s, followed by 1 cycle of 72 °C for 10 min. The PCR products obtained were purified from agarose gel using QIAquick gel extraction kit (QIAgen) and used to synthesize DIG-labelled RNA probes with the DIG-RNA Labeling Kit (Roche).

#### 4.8.2. Staining Procedures

All steps were carried out according to Picchietti et al. [88]. In detail, posterior intestine and liver from adult specimens (*n* = 3) were fixed overnight at room temperature (RT) in 4% paraformaldehyde in 0.01 M, pH 7.4 phosphate-buffered saline (PBS); then, they were dehydrated, embedded in paraffin wax, and cut into 7 μm-thick sections using a rotary microtome. Serial sections were collected on poly-L-lysine coated slides, air-dried overnight at 37 °C, and stored at RT for subsequent investigation. After dewaxing in xylene and rehydration in graded ethanol series, sections were washed with DEPC water before proteinase K (Sigma-Aldrich) digestion. The concentration of proteinase K was titrated for the posterior intestine and liver, and the best results were obtained with 1 μg/mL. The digestion was stopped by immersion in cold DEPC water. Acetylation was performed by incubating sections in 0.25% acetic anhydride in 85 mM Tris-HCl buffer containing 0.2% acetic acid and 0.02 M ethylenediaminetetraacetic acid (EDTA) for 10 min. Following rinses in DEPC water, sections were gradually dehydrated and incubated overnight at 45 °C with the probes (concentrations varying from 0.3 to 0.6 ng/mL), and the optimal one was at 0.45 ng/mL. Subsequently, sections were washed with 2X saline–sodium citrate (SSC) buffer at RT, then with 0.2X SSC at 55 °C for 90 min, and incubated for 30 min with 20 μg/mL RNAase A in 0.01 M Tris-HCl containing 0.5 MNaCl and 1 mM EDTA. Sections were transferred for 1 h to Buffer 1 (0.1 M Tris containing 0.15 M NaCl and 1% blocking reagent), then for 30 min to Buffer 2 (0.1 M Tris containing 0.15 M NaCl, 0.5% BSA and 0.3% Triton X-100). Then, sections were incubated for 2 hrs at RT with alkaline phosphatase-conjugated anti-digoxigenin antibody (Fab fragment; Roche, Basel, Switzerland) diluted 1:1000 in Buffer 2, then washed with 0.1 M Tris containing 0.15 M NaCl and Buffer 3 (0.1 M Tris containing 0.1 M NaCl and 50 mM MgCl_2_). Following staining with nitro blue tetrazoliumchloride and 5-bromo-4-chloro-3-indolyl-phosphate (Roche, Basel, Switzerland), sections were mounted with Aquatex, an aqueous mounting agent for microscopy (Merck KGaA, Darmstadt, Germany), and examined under bright-field illumination. Images were acquired by a Zeiss Axioskop 2 plus a microscope equipped with Axiocam MRC camera and Axiovision software (Carl Zeiss, Oberkochen, Germany).

## Figures and Tables

**Table 1:** The polymeric Ig receptor (*pIgR*) gene size and intron length (nt) in the perciform suborders Notothenioidei, Percoidei, Serranoidei, Scorpaenoidei, and Cottoidei.

Suborder	Species	*pIgR* Gene Size	1st Intron	2nd Intron	3rd Intron	4th Intron	5th Intron	6th Intron	7th Intron
Notothenioidei	*Trematomus bernacchii* *Gymnodraco acuticeps* *Pseudochaennichthys georgianus* *Cottoperca gobio*	8310 8235 9013 5930	628 583 582 596	2372 2866 3080 1519	548 552 538 204	2825 2906 2939 1713	124 124 133 156	146 85 85 129	111 111 111 115
Percoidei	*Sander lucioperca* *Perca fluviatilis* *Perca flavescens* *Etheostoma spectabile* *Etheostoma cragini*	4818 3976 6669 5939 3700	649 590 554 579 595	124 126 125 124 124	211 211 211 153 207	1080 1237 1229 1014 985	161 171 171 143 143	155 137 156 137 145	115 122 115 115 117
Serranoidei	*Epinephelus lanceolatus* *Plectropomus leopardus*	5831 7090	588 545	117 1304	206 183	2969 2135	163 161	93 150	107 118
Scorpaenoidei	*Sebastes umbrosus*	6887	601	779	208	2631	634	133	118
Cottoidei	*Pungitius pungitius* *Anarrhichthys ocellatus* *Gasterosteus aculeatus*	5126 5432 4095	579 595 577	461 593 274	196 205 195	872 1369 1210	136 176 89	167 153 159	96 106 99

## Data Availability

The data presented in this study are available in the Section 4.4.

## Supplementary Materials

The following are available online at https://www.mdpi.com/article/10.3390/ijms23147783/s1.
