# Supplementary material for: Comparative Analysis of the pIgR Gene from the Antarctic Teleost Trematomus bernacchii Reveals Distinctive Features of Cold-Adapted Notothenioidei
Source: Int J Mol Sci. 2022 Jul 14;23(14):7783. doi: 10.3390/ijms23147783 (PMC9321927; doi:10.3390/ijms23147783)
Supplement: Supplementary file 1 [file ijms-23-07783-s001.zip › ijms-1806401-supplementary.pdf]

```
CGCGGTGGATTTAGCAACATAGTTCATGTAGTTCTCCATGTCCACTTGTTGCTGACGGTTGTGGTAGAGGAGCTGTAGAT
GTTGCACTAACATCTGTAGCATGGTCAGTAGCTACTGACAGTAGCTACTGACCATGCTACAGCAGTGAGCAGCAGAGGTG
GGGAGAGGCGGGGCTATGGCTTCATCGTTATCAGGAAATGATCGTATAGGGCGTACACACGGCCGCTTGGCCCCCCAGAG
CGTTCCGTCCGCAGATCTATCCACCTTGGGACCCGTTATCGTTTTAGGGGTCCGTTTCCGTGGTTCCATTACGGCCTTGT
GTGAACGAACGGGCTATACGAAAGAGAAAAGTAGCGGATACAATCCGTTTCGTTCCCGTGTAAACAGCACCTAAGGCTGG
ACCCAATCCAAAGTTCCCAGGCATCGAAATCAAATCATTTCATTGTAGCCTTCCAAATCAATCCTCTGCATTCACGCTGG
GGAAGGCACTCACCATGTGGCTTAGTGGGAGATTAGGTTAAAAAAAGATGGCTAAGTGAAATCATAAAAGAAGGGGATAC
AAGATGAAAGGTGATATGTTTAAATAAACCGGTCCAAAAACCTAGGTTGAAAGATACGTTTTATTTTTAGATGAGAATAT
TGAGCTGTACCAACCTATTCATGTGGCTTTAGCTAATAAGCCTTTTTTCTATAATTTCCATAACTTTATAACACCTTGGA
TATTGTGGAATTTGGTTTAGTCCATACAGGAAGTGACTGAGAATGTAAAGTAAACAAATGTGTCTTTTGGCAGAAATGCA
ACAATTTGCCCCCTACTCCTTAAAAGAGGAGAGCTGATGTCATAGAAAGTGTGGTTACATATTTATGCTACAGAAAGTGA
TAATGATAAAAGTATTCCTTACTATCTAACTGGCACTTACATGGTTACATCTGGAACATCTGGACCCTGCTCTTGGTCAC
ATGACTTTGGGGGTTATTGCCTTTGCGGAACTTAGTTTCTGTTTTA
                                                 [Leader
CAGAAGGGAGATTCACTTGGAAAACCTCAAAGTGAAACATGCTGAAGCCCTTTCTACTCACCCTCAGCCTGTTACCATGG
                                         M  L  K  P  F  L  L  T  L  S  L  L  P  W
ATCCCAGGTAATAACTGAGCTCATATGCTTGAAAATAGTTCTTAGGATGTTTAATGTCTATGATGACCAAAAATCGCTTT
 I  P
TAAATGTGCTTTATAAATAAAACTTGATTTGATTTGATGATTCCATGGCTTCAACAGTGTGCTTCAGGCAAATGCACATA
GGCAATATGTTGCAATGACAACTCATGCTTCACTTCCCTTTACTATTTATTACTTTTTTGTGTTAATCATTTGCTGAAT
ACAAACTATAAAGGCTGGTAATGCTATTTTCCGTGGCAGCCAGAGAACGCTTTAGTGTTTTGTTTTTTAAATCTTCTAAG
CTTCTGTTCTGCATGCCCCTTAGCAAAGAGTATGTTTGATGTTTATTTTGTCAAGGCTCCAAATGCCTGTTATATTTGTT
CCCCCGACTCTCTATTATACTGTAGAGGGATAATCTCATTTTAAAATATGGAAAGGGAAGAGGAAGTAGAAAAGTGTTT
TGTTAGAATGGAAAACAAAACCATGAATGTGGTACAAGTATTGCTCAAGGCCACTTTTACCCGGCCGTTCCTCAAAACAT
AGATTAGAAACCTTTGAGACATGGCTGACACTCCAAAATAGAGCTCCACATTAAATAATCTACACTTTCCATCAG
                                                                           TTTTC
            ]                                                              V  F
CTCTGCGGGACAACCACCGAAGAAGAGCTCTCAATCATGGAGGGTCAAAGCCTCACCGTCCCGTGTCACTATGAGCCCCA
 L  C  G  T  T  T  E  E  E  L  S  I  M  E  G  Q  S  L  T  V  P  C  H  Y  E  P  Q
GTATGCCAGCTATGTAAAGTACTGGTGCCGGGGAACGATGAGGGAGTTCTGCTCCAGCCTAGCTCGGACCGATGAGTCCC
  Y  A  S  Y  V  K  Y  W  C  R  G  T  M  R  E  F  C  S  S  L  A  R  T  D  E  S
ATTCTGCCAATCCGTCTGAGAAGAAAGTGAGACTCTTTGACGACCCGGTGCAGCAGGTGTTCACTGTTGCCATGAGCAAC
H  S  A  N  P  S  E  K  K  V  R  L  F  D  D  P  V  Q  Q  V  F  T  V  A  M  S  N
CTGAGGGAGGAGGACTCTGGGTGGTACATGTGTGGCGTGGAAATTGGTGGCCTGTGGAGCGCTGATGTTGTCACTCACAA
 L  R  E  E  D  S  G  W  Y  M  C  G  V  E  I  G  G  L  W  S  A  D  V  V  T  H  K
AAACATCAATGTCATTCATGGTGAGTAATGGATGGATTTTAAATGTGATCTATCCCCATTCAAAAATGATTTTCACATAT
  N  I  N  V  I  H
GATAATAATGTGTACTTATCATGTGTTATGATCTGAACATAGCACTGTATAATTATAGCTGTAAGCACTAATAAATAAAT
GTAATGCTTTTTATAACAATATTAATACTTTTAATCACTGACACTGTTTTTCCTTGTTTTTGCAAGGATCATAATGTATT
ATAATTCCCTTATGATACATTCTCATCACTGTTTTAATGCATTATTATTCAAGAATTTCCTTACTTAACTTCAAAGCATC
AAATGACCATGTTGAGCAATTCATAACCACATATTAATCCATTATAACGCTAATGATAATGCAATAAAGCAATGTGAATT
GTATGTTGGTAAATGTACAGTACTGTAAGCAGATTATAACGCATTATAAATAAATGTATAATACAAAGTATAGACATACA
AAGTGTTACTGAAAACAAAATAATTTCAAATGCTTAGATTACATATATGAATAAACAGTATTTATATGAATAAATATATA
TATATATATTTAAGTTTGTATTATATGAAAGGTGGCCTATTATGCAAAATCCACTTTTATACATAAATATGTGTCCCCTC
CGTGTAAAGAGATTAAGAAAATTTCAGGAAAAAAGATTCTCTCTCTTTTTGTCCTGAACCATCTACATAAAAACCTGTCT
GACAATGAGCTGATCAGATTTTGGCCACGGTGTGATTTCACAATGATGTTTTGGAGCATCCCATAACAAAGTCGTAAATC
GGTATAGCGGCAGCTGGGAATAGACAGCAGCCGGACCTGCGCATGCAAGTTTTCTCGTTAGGATAGTCGGATGTTACTCT
TTGCCCTCCAGAGGGTGAGAAAATAAGTGTGAATGCCTTCTATTCTGTGGGTAAACAAATGAATCAAGTCTAGGCTCCTC
CAGTCTCGACAGGAGTCAGCTGAAAGGTCATGCACAGTTCCAACAGTGTAAGATCACAGTGGCACACACTATTAAGCCGT
ATTTTTCCCAACAGCAACCAGCAAAGATAACACCTTATCACCTGAATCTCCTCCTAGAGCACCATTATGTTTTTTTAAAC
TAAATATCTCGCAGAGGGGCGTGGCCACCAGCAGCTCATTTGCATTTAAAGCTGCAGACACAGAATCAGCCCTTTTGAAA
CAGGACTGAAACAGAGGGGTTTATGGGATGTTACAATGCATGATCTGTTTGGTATTTTGAGCCAGACACTTCAGAGACAT
GTTTTTTATTTATCTGAGACCCATAATATATTGTTGAAAAATAGTTTAATAGGGTACCTTTAAGGCATGTCAATGGAACT
TAATTATACCACTTTGGATGTAAACAGGTGTACATTAATCCTTGAACATGTAATAACAATCTAGACAAATCCCTCCAAAG
GAAGACTTATAAACAGCTGAATACATGCCCATAGCTTCTGAGATCACATGTGCAAATTCAGGCTACCAATTGCTGTGCAT
ACAAAAGGGACTTTGTATCTTTTTCCCTCCTCAAAGCAGGAACTCTATCTTCTAAAGGTGAGAGCTCTGCACAGGGTGAT
CAGAATAGTCCAATTTGGCCTTACCCTACTTACAGTATGACCTGCCTATTTTCCTACAGTGACAACCTGGTCAAATCTCA
ACAACGTAGGTTATTGAGGTTCCATTGTAGCGTACACATTATGGAGTTTTTGCACGGACATTGTTGCACCTTTAAAGAAA
TTGCATCTACATTGGGAGGAAGCTGAACTTTTTTCCAATACCGTACCGAAATACTAGTAATTTTGCATCAGTTCACCTTA
AAGGGGCCCTATTGTGCTTTTTCTGGTTTTCCCTTTCCTGTAGTGTGTTATGTCAATGGTCGGCAAAGGCTAAAATCCCA
AAGTTTCCTCCAGAGGGAGTTTCTGTCCCGCACACTCACCACCTGCCTGAAACGCCTCCATTGAGCTAATTTCTTTACTT
CCGTAACATAGTGACATCACTGTAACACTTGCGCTTCTATTAGCTAGTGCTCCAACACTACATGAAAGGGCCATGACATC
TCTGAGCAGTTGACCAATCACAACAGAGCTGGCCAGCTAACCAATCAGAGCAGACTGGACTCTGGTTTTAGACAGAGGGT
GAAAAGAGGTGCTGCAGCACAGGCAGTATGATAACAATAAAGACCTTTTTTTAACATTAAAGCATAGAGACATGTCACAG
TAGAGACACAAAATACAATTATGAAAGCTGAAAATGAGCATAATAGGGCCCCTTTAATATCTGTAAGTTTTGGGTCGGTT
GCCAAGTCCCTTAAGTCAAAGATAATGTTTTTTGTTTTAGACACGTTCATCAAGTTTATTCCTTTCCTGCAGGTATGACA
                                                                          G  M  T
GTGGAGGACAGGCGCCTGAGTGGGGAAGAAGGGAGTAGTGTCACAGTTGAATGCCAATACAGCGAGAGATACAGGTATTT
 V  E  D  R  R  L  S  G  E  E  G  S  S  V  T  V  E  C  Q  Y  S  E  R  I  R
TATTGCCACATACATCATTTTACAATTGTGTCAGCTGTATTTACCATCAGACCATGTAGAGTCCTCACTTTTAAGTCGAC
ATTTTCTAGAGCATCAGCAAAGTTAGGAAAGATTGTTATTTGTTCAATATATTTGTATATACTTTTTATTATTGACTTAG
TTTGTCTATACATGTTAAAAATACAAACTCTTGTTTTACATATAGTTAAAAAAAGGGGTAAAGTGTGTATGTAAAGTCAG
AATAATGCTGATTGAAATAGTTAAGCATACTTCATGAAAGGCAGAAACACAGAGGGATATTATATATAAATATACAAACA
CACATAAATACATACACACATACTCATATGCATATTCACTAAAGATAAGTTCATACATGTGCATACATACAGTAAGTAAA
ACAACCAAAAACAACCAGCATACAGTAAAGAAAAGAGAAAACGGATTAATATTTTGGCCCTGCTTCAATTTGTTCAATAT
CTGCCATTCTTGCTAAACTGTGATGGACACTCGCTGACTTCCCTGTGAATGTTTGCCCACAGAGAGAGTGAGAAGAAGTG
                                                                    E  S  E  K  K  W
GTGTCGGAGTGGAGACTCGAGCTCCTGTCTGTTGGCGGGATCTGAAGGGAGCAACGGAAACTCTTCAGTGGACATCAAAG
  C  R  S  G  D  S  S  S  C  L  L  A  G  S  E  G  S  N  G  N  S  S  V  D  I  K
ATGACAGATCTGGGTCTTTCACCATAACCTTTAAGAAGCTTCAGATGAGGGATACAGGCTGGTACTGGTGCTCTGCGGGA
D  D  R  S  G  S  F  T  I  T  F  K  K  L  Q  M  R  D  T  G  W  Y  W  C  S  A  G
CTTCAAAAGATGCCCGTGCATGTGCAGGTCGTACCACGACCCATGACTAGTAGGTTGACAAATACAAATGAATATATCAA
 L  Q  K  M  P  V  H  V  Q  V  V  P  R  P  M  T
GTTAAACTTGATATGAACACAGGATAGAATTTCAAATTCTTCTTCTCTCCTACCTGGGCAGGCTCCATCTTACCTTAAAG
AACTCATTATACCCTACTGTCCTACTAGGGCATTGCGTTCCAAGAATGCACGGTTGTTGGTTGTTCCTAGAGTCTCTAAA
AGTACAATGGGAGCCAGAGCCTTTTCTTATCAAGCTCCACTTTTGTGGAATCAGCTTCCAGTTTGTGTTCGGGCGGCAGA
CAGCCTATCCGTTTTTAAGAGTACCCTTAAGACCTTCCTTTTTGATAAACCTTATAGTTGATAAAATAATCTAATAAGAG
TGCATTGACGTAGACACGTTGTGCCTCTTCACCTATACATCTTAGTTCTTACTCTTTTCCTTCACTGATGGTATAGATGT
AATTTGGTTGTGTGTGTAGACGGTGGAAGCAGGGAGAGGCATGTACGTGCCTTGATCCCTGCCGAGTCGACGCACAACTG
TTAGTGTGCGTGCGTGTCAGTGCCAGAAACACTTTTTAGGAGCAATACGCTCATGGTATAGATGTGGCGCATACATCTTA
GTTCCTACTCTTTTCCCTCACCCTATCCTAAGGGAGTGCATGTACGTACGCAGACACTTTATGCCTCTCCACCTCTGTTT
CTTACTTCTTACTGGCTACCTCTTTCCTTCAACCTATCCTAAGGGAGTCCAAATTTCTTTTTGGTGTCTATCTATGCCGG
GATCCTGAGTCGTGGCTGATCCTGTTGCTGCGGTCCTGTGTCCTGGATCCTGAGTCATGTGTCCTGGATCCTGCATCCTG
AGTCCTGGATCCGGAGTCCTGGATCCTTAGTCCTGAGTCCTGGATCCTGTGTCCTGGATCCTCTATCCTGAGTCCTGGAT
CTGAGTCCTGGATTTCGAGTCCTGGCTGAACCTGTCACTGTGGTCCTGCCTGACTCTCATCATATACTTCCCTGATGGCT
CCCACAGGATTGTTGACATCCTCGTGGATTCATCTTCTTATTATAGACACATGCATTTCCAAACATTTGGTCTACCTATG
CTGTAAAATGTATTATCTCTTCGATTCACACATGGCATCTATTGCACGTCTGTCCGTCCTGGGAGAGGGATCCCTCCTCT
GTTGCTCTCCCTGAGGTTTCTCCCATTTTCCCCTTTAAAACTGTGGGGTTTTCTTTGGAAGTTTTTCCTTGTACGATGTG
AGGGTCTAAGGACAGAGGGTGTCGTATTGTCATACTGATATTCTGTACAAACTGTGAAGTCCACTGAGACGAATGTAACA
TTTGTGATATTGGGCTATATAAATAAACATTGATTGATTGATTGATTGATTGATGAAGGAATGTCTGTAATTCATTATAC
ACTTCCAATGTATTATTATCTGCGTAAAGAATGGTGTAATTATAAGTACTTGCTTTATATCAATAATCATTAGATATTTT
TTTCAAAAGGACATTAAAGATCAAGTATCATAATGCTTTATAACTGCAGTTGATTCACTGACCCTTATAATTTGCCAGAA
TCATAGTGTATTCTTTTCTAATATGTGTAATCATTTTATTGATGACATGGTTTGATTTAGTCAGTAGTAGTAGTTACTAA
ATCAACTTTTTTTGTTGTTGTTCTGGCATAGATTACACTTTACTCATAACACATTATTTCAGGAATTATAATGCATTGCA
AAAGAGATGTATGACAAAAATTTGAATTATACACTATAATCCTTGGGGAAAAAGGCAGAGAGAAACCAGCATTAATGTAT
TCAGTATTTAATATTAAGTGTCAAAATCTTGCAATCAATGGGAATCTGTTGGCGAGTCATTTTGAGAAACCAAAACAATG
TGCTGAAAGACACTACATTTATCAAGAGAACCCAAATAGTTGGTTAATAATTCTCTGTGAGTGTTCCACTGTGAGAAACC
ACGTTCACATGTGTACTTGTACCTCATGTCAAATGTATAATACACTGTTTTGCCCATAAAGTTGGACACATTTTGTTTTC
AGGCACATTCCCCTTTTTATTCCTATTTACACACACAATTTATGGGCCACAGTGTATGTAACATTTAAGCTTTAATATGT
CTAAAAACATCTCAACCTACAGTATGACATATATATTGTTAGGTTGTGTACTAACAATATCCCCAATGTTTCCAATCGCT
TTCAAACTTAGAGAAGTCCGTCATTTTATTCAAGGTTATGGTCCGTCTCATTTTCTCGCCTTTAATATCGACGTATCCCA
TTGTACATGGTAAGCGTTGTGGTTGTCTGCCAGAAGTGATCATACCTTTCTCTGTGGGTTACTCAGCTGCTAAGCCATGA
ACCCCGCCAAGCCAACCTAAATCTGACAAGATCAGATTTCAGCCTGCGATTTCATTCTGGGAGACTTCTATTAAATGTTG
AGCCTAGTCTGTGGATATCACAGAAATCAGTGTACTGTGGCGAGTTGTTGTTTAGATCTGACTTGCCAATTGAAACACCA
AATTGTTATAAAATAGTATTTAAACTAGGCATAATAGGTTTGAAAATAATCACAATAATGTAACTATGCAACCTTTTTCC
CCACGTCATATTGCGGCTGCTGATATAAAAAGCAGCCTACAGCAGCTTTGAAGATCAATATGTGTTTGTGACAGATTGCA
GGGGCTCATGGGTATCCAGCTGCACAAGGTATCGATTATCACGAGTCACTTATCAAGAATATTGATCTTAAGAACTCTGT
GGATGCTGCTACAAACACTCTTTTTCTTATGATAGTACATACTGTTTCAGCTGTACCATCTCTTTCTTTAGCAGCAGTGT
                                                                          T  A  V
CTGTGACATCCCAACCTCAGACTGTTGCAAATCCTCTTCCCCCAAACCCATCACTAAGGAGTCGTGGAGCGGTCACAAG
S  V  T  S  Q  P  Q  T  V  A  N  P  L  P  P  K  P  I  T  K  E  S  W  S  G  H  N
TAAGAGTGAAGCACTACTGTTAATGAGAAATCTGTCATCATGTTGGACCTCATGTTAATATGTTGCAATTCAGGCCTGGG
CTGCTATTGAAGCTGCTCTGATAACTGTGTGTTTGTGTCTTAGTTTTAGTCACATCTTGGGGTCCTTGCTGGTGTGTGGT
                                              F  S  H  I  L  G  S  L  L  V  C  G
TCTGTAATCCTTGTGGGTTTGGCCATAGTGGCGAGGAAGTGGTGGAAACGACACAGTAAGGCAACTTCTTATTTATTTGA
 S  V  I  L  V  G  L  A  I  V  A  R  K  W  W  K  R  H
ACATTACTGTACATTTTAGCCGTATCTCCTCTATAGTTCTATGAAACATGTATTAACTATATCAGAGTAAGATATCTGCA
TTATCATGTGTGGCTTAAAGACCCTGAATATCCATTTCTAGAGCAGGATCCTATGCTGAGACAACTTAATGGGATGAACG
                                            K  Q  D  P  M  L  R  Q  L  N  G  M  N
CAAGGCGAAATGTAAGCCCCCGTTATAATATCTGTCACTATTTTATTGCACAAAATATAAGCACCATTATCAAGGATATG
A  R  R  N
CTTTAGCGATTATTGTTTAATTTATCTTTCTGTCTCCCACAGGAGTACAGCGATGTGGGTTGTGACCTGCAGAATGCTAC
                                             E  Y  S  D  V  G  C  D  L  Q  N  A  T
TGTCGTTTTCGTTAACAAGGATTCTCAGGATGTACATATGTACTGAGCTTTGTCAGTGCAATGTTTTATTACTGCTCTTT
  V  V  F  V  N  K  D  S  Q  D  V  H  M  Y
GGGCTGAATGTATTTTCTTGTTACTTCACTGCACACAGCAGATACTTTATTTTTTATGTCTTTTAAACATGCCAGCACAT
TTAAAGGCGCTTATTAATATTTTTGGTCAGATGTACTGTACCTACAGTACCACTAACAAACCTGGGCTCATGTTATATGT
TTTATAATATTTCTTTTATGGAATGTGGGGAACTTTCGCAAATTGGTGGAGTTATATTCTAAAGTTTAACACATTTCAGT
TGATTAGGACGTTTTTGTTATTATTTTCATAAATACAATTAAATCAATAAATACATATTTGATTGATTATT
```

Figure S1. Complete sequence of *Trematomus bernacchii* polymeric Ig receptor (*pIgR)* gene. The deduced amino acid sequence is reported in one-letter code. The sequence comprises a promoter region, including the leader peptide (in cyan), a region encoding the D1 domain (in green), the D2 domain (in yellow), the extracellular membrane proximal domain (EMPD, in magenta), the transmembrane domain (TM, in dark green), the cytoplasmic tail (in blue), and the terminal sequence (in dark yellow). The stop codon is in white and underlined, polyadenylation signals are indicated in yellow. The intron sequences are shaded in grey; the donor and acceptor sites are depicted in red. The region containing the CpG island (CGI), located upstream of the 5′ end of *pIgR* gene, is highlighted in purple.

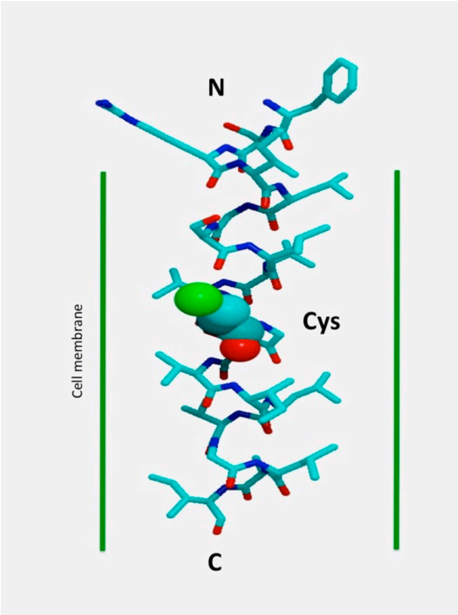

Figure S2. 3D molecular model of the transmembrane helix of *T. bernacchii* pIgR built with the Phyre2 tool (http://www.sbg.bio.ic.ac.uk/phyre/html/). The cysteine residue, located in the middle of the sequence with its side chain pointing out of the transmembrane helix, is shown. The N- and C-termini are indicated at the top and bottom, respectively.

Signal peptide

Trematomus bernacchii	MLKPFLLTLSLLPWIPV
Trematomus loennbergii	V
Dissostichus eleginoides	PWIPA
Dissostichus mawsoni	
Notothenia coriiceps	LPWIPV
Harpagifer antarticus	V
Gymnodraco acuticeps	MLKPFLLTLSLLPWIPV
Pseudochaennichthys georgianus	MLKPFLLTLSLLPWIPV
Chionodraco myersi	V
Chaenocephalus aceratus	V
Chionodraco hamatus	SLLPWIPV
Cottoperca gobio	MLQPFILALSLLPWIPA
Sander lucioperca	MLQPFIITLSLLPWFPA
Perca fluviatilis	MLQPFLIVLSLLPWFPA
Perca flavescens	MLQPFIITLSLLPWFPA
Etheostoma spectabile	MLPPFIIALSLFPFFPA
Etheostoma cragini	MLQPFTITLSLLPFFPA
Epinephelus coioides	MRRLFILTLSLLPWIPA
Epinephelus lanceolatus	MRRLFILTLSLLPWIPA
Plectropomus leopardus	
Sebastes umbrosus	MLQPFILALILLPWIPA

```
Pungitius pungitius                                                   MRKLFTLALTLFPWISG
Anarrhichthys ocellatus                                               MQQPFILALSLLSWIPA
Cyclopterus lumpus                                                    MLRPFLLALSLLPWIPA
Liparis tanakae
Gasterosteus aculeatus                                                MRKLFTLALTLLSWIPG

                                         /D1               L              S       S
Trematomus bernacchii               FLCGT-TTEEELSIMEGQSLTVPCHYEPQYASYVKYWCRGTMREFCSSLA
Trematomus loennbergii              FLCGT-TTEEELSIMEGQSLTVPCHYEPQYASYVKYWCRGTMREFCSSLA
Dissostichus eleginoides            FLCGT-PTEGELSVMEGQSLTVPCHYEPQYASYVKYWCRGKMREFCSSLA
Dissostichus mawsoni                FLCGT-TTEEELSVMEGESLTVPCHYEPQYASYIKYWCRGKMREFCSSLG
Notothenia coriiceps                FLCGT-TTEEELSVMEGQSLTVPCHYEPQYASYVKYWCRGTMREFCSSLA
Harpagifer antarcticus              FLCGT-TTEEELSVMEGQSLTIPCHYEPQYASYIKYWCRGKMREFCSSLA
Gymnodraco acuticeps                FLCGT-TTEEELSVMEGQSLTVPCHYEPQYASYIKYWCRGKMREFCSSLA
Pseudochaennichthys georgianus      FLCGT-TTEEELSVMEGESLTVPCHYEPQYASYIKYWCRGKMREFCSSLA
Chionodraco myersi                  FLCGT-TTEEELSVMEGESLTVPCHYEPQYASYIKYWCRGKMREFCSSLA
Chaenocephalus aceratus             FLCGT-TTEEELSVMEGESLTVPCHYEPQYASYIKYWCRGKTREFCSSLA
Chionodraco hamatus                 FLCGT-TTEEELSVMEGESLTVPCHYEPQYASYIKYWCRGKMREFCSSLA
Cottoperca gobio                    LLCST---EGELSVLEGQSLTVPCHYEPQYASYVKYWCRGKTKEFCSSLA
Sander lucioperca                   FLCRV-TTEGEHAVMEGQPLTVPCHYGPQYAGYVKYWCRGKMREFCTSLA
Perca fluviatilis                   FLCRV-TTEGEHAVMEGQPLTVPCHYGPQYAGYVKYWCQGKMREFCTSLA
Perca flavescens                    FLCRV-TTEGEHAVMEGQPLTVPCHYGPQYAGYVKYGCRGKMREFCTSLA
Etheostoma spectabile               FHCGV-TTEGEHTVMEGQHLTVPCHYGPQYAGYVKYWCRGKMREFCSSLA
Etheostoma cragini                  FLCGV-TTEGEHAVMEGQHLTVPCHYGPQYAGYVKYWCRGKMREFCTSLA
Epinephelus coioides                VLCKV-TTEGELSIMEGQSLTIPCHYEPQYASYVKYWCQGKTREFCTSLA
Epinephelus lanceolatus             VICKV-TTEGELSIMEGQSLTIPCHYEPQYASYVKYWCQGKTREFCTSLA
Plectropomus leopardus              FLCGT-TTEEELSIMEGQSLTVPCHYEPQYASYVKYWCRGKMKEFCTSLA
Sebastes umbrosus                   FHCRV-TTEGDIAVMEGQSLTVPCIYEPQHASYVKYWCRGKMKAFCTTLA
Pungitius pungitius                 FLCQPAFTGGELSVMEGQSLTVPCHYESQYAGHVKYWCRGKVRGFCTSLA
Anarrhichthys ocellatus             FLCRV-TTEGELTVMEGQSLIVPCHYDPQYAGYVKYWCRGNTREFCTSLA
Cyclopterus lumpus                  FLCRV-TTEGELAVMEGQSLTVPCHYDPQYAGYVKYWCRGKMREFCTSLA
Liparis tanakae                     MDPRV-TTDAELAVMEGRSLTVPCHYDPQYAGYLKYWCRGKMREFCTSLA
Gasterosteus aculeatus              FL-----SEAELSVMEGQSLTVPCHYEPQYAGYVKYWCRGKMRGFCTSLA

                                                                               L
Trematomus bernacchii               RTDESHSTNPSE-KKVRLFDDPVQQVFTVAMSNLREEDSGWYMCGVEIGG
Trematomus loennbergii              RTDESHSANLSE-KKVRLFDDPVQQVFTVAMSNLREEDSGWYMCGVEIGG
Dissostichus eleginoides            RTDESHSANLSE-KKVRLFDDPVQQVFTVAMSNLREEDSGWYMCGVEIGG
Dissostichus mawsoni                RTDESHSANLSE-KKVRLFDDPVQQVFTVAMSNLREEDSGWYMCGVEIGG
Notothenia coriiceps                QTDESHSANPSE-KKVRLFDDPVQQVFTVAMSNLGEEDSGWYMCGVEIGG
Harpagifer antarcticus              RTDESHSANPSE-KKVRLFDDPVQQLFTVAMSNLREEDSGWYMCGVEIGG
Gymnodraco acuticeps                RTDESHSANPSE-KKVRLFDDPVQQVFTVAISNLMEEDSGWYMCGVEIGG
Pseudochaennichthys georgianus      RTDESHSVNPSE-KKVRLFDDPVQQVFTVAMSNLREEDSGWYICGVEIGG
Chionodraco myersi                  RTDESHSVNPSE-KKVRLFDDPVQHVFTVAMSNLREEDSGWYMCGVEIGG
Chaenocephalus aceratus             RTDESHSVNPSE-KKVRLFDDPVQQVFTVAMNNLREEDSGWYMCGVEIGG
Chionodraco hamatus                 RTDESHSVNPSE-KKVRLFDDPVQQVFTVAMSNLREEDSGWYMCGVEIGG
Cottoperca gobio                    RTDVTRSANTAE-EKVGIFDDPVQLVFTVTMSNLREEDSGWYMCGVEIGG
Sander lucioperca                   RTDEPRSANQAE-EKVSIFDDPVQLVFAVTMSNLKEGDSGWYMCGVEIGG
Perca fluviatilis                   RTDEPRSANPAE-EKVSIFDDPVQLVFTVTMSNLKEGDSGWYMCGVEIGG
Perca flavescens                    RTDEPRSANPAE-EKVSIFDDPVQLVFTVTMSNLKEGDSGWYMCGVEIGG
Etheostoma spectabile               RTDEPRSANPAE-EEVSIFDDPVQLVFTVTMSNLKEGDSGWYMCGVEIGG
Etheostoma cragini                  RTDEPHSANSAE-EKVSIFDDPVQLVFTVTMSNLKEGDSGWYMCAVEIGG
Epinephelus coioides                RTDEPRSADPAE-KKVSIFDDQVQLVFTVTMNNLKEGDSGWYMCGVEIGG
Epinephelus lanceolatus             RTDEPRSADPAE-KKVSVFDDQVQLVFTVTMNNLKEGDSGWYMCGVEIGG
Plectropomus leopardus              RTDEPPSADSAK-KKVSIFDDQVQLVFTVTMNDLKEGDSGWYMCGVEIGG
Sebastes umbrosus                   RTDETRSADPAEEKKVSIFDDPVQLVFTVTMNNLKEDDSGWYMCGVEIEG
Pungitius pungitius                 RTDP----AIAAAGKVSISDDRVQLVFTVTMSDLKEGDSGWYLCGVEIGG
Anarrhichthys ocellatus             RTDS----ANPAAGKVSIFDDPVQQVFTLTMSDLKEGDSGWYMCGVEIGG
Cyclopterus lumpus                  RTDP----AHPAARKVSIFDDPVQQVFTVTMGDLKETDSGWYMCGVEVGG
Liparis tanakae                     RTDP----ATPAVKTVSLLDDPVQQVFTVTMDDLKEADSGWYICGVEVGG
Gasterosteus aculeatus              RTDT----ANPAAGKVSMSDDPVQLVSTVTMSDLKEGDSGWYICGVELGG

                                                  /   /D2            L
Trematomus bernacchii               LWSADVVTHKNINVIHGMTVEDRRLSGEEGSSVTVECQYSERYRESEKKW
Trematomus loennbergii              LWSADVVTHKNINVIHG                                KKW
Dissostichus eleginoides            VWSADVVTYKNINVIHGMTVENSRLSGEEGSSITVECQYSERYRESEKKW
Dissostichus mawsoni                LWSADVVTHKNINVIHGMTVENSRLSGEEGSSVTVECQYSERYRESEKKW
Notothenia coriiceps                LWSADVVTHKNIKVIHGMTVENSRLSGEEGSSVTVECQYSERYRESEKKW
```

Harpagifer antarcticus	LWSADVVIYKHIKVIHG KKW
Gymnodraco acuticeps	LWSADVVTHKNIKVIHGMTVENSRLNEEEGSSVTVECQYSERYRESEKKW
Pseudochaenichthys georgianus	LWSADVVTYKNIKVIHGMTVENSRLSGEEGGSVTVECQYSERYRESEKKW
Chionodraco myersi	LWSADVVTYKNIKVIHG KKW
Chaenocephalus aceratus	LWSADVVTYKNIKVIHG KKW
Chionodraco hamatus	LWSADVVTYKNIKVIHGMTVENSRLSGEEGSSVTVECQYSERYRESEKKW
Cottoperca gobio	VWSADVVTYTNIRVIHGLTVVNSRLSGGEGSSVTVECHYSERFRESEKKW
Sander lucioperca	AWSADDVAYTNIKVIHGMSVVNSRLIGEEGSSITVECHYSERYRESEKKW
Perca fluviatilis	AWSADDVAYTNIKVIHGMSVVNSRLIGEEGSSITVECHYSERYRESEKKW
Perca flavescens	AWSADDVAYTNIKVIHGMSVVNSRLIGEEGSSITVECQYSERYRESEKKW
Etheostoma spectabile	MWTADDVAYTNIKVIHGMSVVNSFLIGEEGSSITVECHYSERCRESEKRW
Etheostoma cragini	IWSADDVAYTYIKVIHGMSVVNSFLIGEEGSSLTVECHYSERCRESEKRW
Epinephelus coioides	VWSADDVAFTNIKVIHGMSVVNSRVSGEEGSSLTVECHYSERYRESEKKW
Epinephelus lanceolatus	VWSADDVAFTNIKVIHGMSVVNSRVSGEEGSSLTVECHYSERYRESEKKW
Plectropomus leopardus	VWHSDDVAFTNIKVIHG KKW
Sebastes umbrosus	VWHSDDVAFTYISVIHGMSVVNSRLSGEEGSSVTVEC**NYS**ERYRESEKKW
Pungitius pungitius	AWTADVVTQTYINVIHGMSVVNSRLSGEEGSSVTVECHYSEKYRDSEKKW
Anarrhichthys ocellatus	VWTRDVVASTYIKVIHGMSVVNSRLSGEEGSSVTVECRYSERYRDSEKKW
Cyclopterus lumpus	VWTADVVAYTYIKVVHGMSVVNSRLSGEEGSSVTVECHYSERYRDSEKKW
Liparis tanakae	GWTPDVVAHTYIKVVH
Gasterosteus aculeatus	AWTADAVTETYIQVIHGMSVVNSRLSGEEGSSVTVECHYSERYRDSQKKW

	**S S L**
Trematomus_bernacchii	CRSGDSSSCLLAGSEGSNG**NSS**VDIKDDRSGSFTITFKKLQMRDTGWYWC
Trematomus loennbergii	CRSGDSSSCLLAGSEGSNE**NSS**VDIKDDRSGSFTITFKKLQMRDTGWYWC
Dissostichus eleginoides	CRSGDWSSCLLAGSEGSNEDSSVDIKDDRSGSFTITFKKLQMRDTGWYWC
Dissostichus mawsoni	CRSGDWSSCLLAGSEESNEDSSVDIEDDRSGSFTITFKKLQMRDTGWYWC
Notothenia coriiceps	CRSGNWSSCLLAGSEGS**N-DS**SVDIKDDRSGSFTITFKKLQMRDTGWYWC
Harpagifer antarcticus	CRSGDWSSCLLAGSEGS**N-DS**SVYIKDDRSGSFTITLKKLQMRDTGWYWC
Gymnodraco acuticeps	CRSGDWSSCLLAGSEGS**N-DS**SVYIKDDRSGSFTITFKKLQMRDTGWYWC
Pseudochaenichthys georgianus	CRSGDWSSCLLAGSEGS**N-DS**SVYIKDDRSGSFTITFKKLQMRDTGWYWC
Chionodraco myersi	CRSGDWSSCLLAGSEGS**N-DS**SVYIKDDRSGSFTITFKKLQMRDTGWYWC
Chaenocephalus aceratus	CRSGDSSSCLLAGSEGSNG**NSS**VDIKDDRSGSFTITFKKLQMRDTGWYWC
Chionodraco hamatus	CRSGDWSSCLLAGSEGS**N-DS**SVYIKDDRSGSFTITFKKLQMRDTGWYWC
Cottoperca gobio	CRSGDWSSCLSTGSEGSYDDTSVAISDDRTRTFTVTLKKLQMRNTGWYWC
Sander lucioperca	CRSGDWSSCLLTGSDGSYEDTSVAISDDRTRTLTITLKKLQMRDTGWYWC
Perca fluviatilis	CRSGDWSSCLLTGSDGSYEDTSVAISDDRTRTFTITLKKLQMRDTGWYWC
Perca flavescens	CRSGDWSSCLLTGSDRSYEDTSVAISDDRTRTFTITLKKLKMRDTGWYWC
Etheostoma spectabile	CRSGDWSSCLLTDSDGRYEDTSVAISDDRTRTLTITLKKLQMRDTGWYLC
Etheostoma cragini	CRSGDWSSCLPTGSDGRYEDTSVAISDDRTRTLTITLKKLQMRDTGWYLC
Epinephelus coioides	CRSGDWSSCLLTGSEGSYEDTSVAISDDRTRTFTITLKKLQMRDAGWYWC
Epinephelus lanceolatus	CRSGDWSSCLLTGSEGNYEDTSVAISDDRTRTFTITLKKLQMRDTGWYWC
Plectropomus leopardus	CRSGDWSSCLLIDSEGSYEDTSVAISDDRTRTFTVTLKKLQMRDTGWYWC
Sebastes umbrosus	CRSGDWSSCLLTGSEGSYEDTSVAIRDDRTRTFTITLKKLQMRDNGWYWC
Pungitius pungitius	CRIGDWSSCLLTGSEGSYDDTSVAIRDDRTRTFTVTLKKLQMKDTGWYWC
Anarrhichthys ocellatus	CRSGDWSSCLLTGSEGSYEDTSVAISDDRTRAFTVTLKKLQMSDSGWYWC
Cyclopterus lumpus	CRSGDWSSCLLTGSEGSYEDTSVALRDDRTRTFTITLKKLRMRDSGWHWC
Liparis tanakae	
Gasterosteus aculeatus	CRTGDWSSCLLTGSEGSY**NDT**SVAIRDDGSRTFTVTLKNLQMKDTGWYWC

	**/EMPD**
Trematomus bernacchii	SAGLQKMPVHVQVVPRPMTT-VS---VTTQPQTVANPLPPKPITKESWNG
Trematomus loennbergii	SAGLQKMPVHVQVVPRPM
Dissostichus eleginoides	SAGLQKMPVHVQVKPRPMTTAVS---VTSQPQT-ANPLPPKPITKESWSG
Dissostichus mawsoni	SAGIQKMPVHVQYILLS---------VCDIPTSDCKSSSPKPITKESWSG
Notothenia coriiceps	SAGLQKMPVHVQVKPRPMTT-VS---VTSQPQTV PKPITKESWSG
Harpagifer antarcticus	SAGLQKMPVHVQVKPRPM
Gymnodraco acuticeps	SAGLQKMPVHVQVKPRPMTMS-----VTSQPQTVANPPPPKPITKESWSG
Pseudochaenichthys georgianus	SAGLQKMPVHVQVKPRPMTT-VS---VTSQPQTVANPLPPKPITKESWSG
Chionodraco myersi	SAGLQKMPVHVQVKPRPM
Chaenocephalus aceratus	SAGLQKMPVHVQVKPRPM
Chionodraco hamatus	SAGLQKMPVHVQVKPRPMTT-VS---VTSQPQTVANPLPPKPITKESWSG
Cottoperca gobio	SAGQQKMPVHVQVTPRP-TTTVS---VTSPPQSIAYLPPPKPITKESGNN
Sander lucioperca	SAGQQQIAVHVLVTPRP-TTAVSVTSTPTTSQSVAYLPPPKPITKESWNS
Perca fluviatilis	SAGQQQIAVHVLVTPRPTTAAVSVTSTPKTSQSVAYLPPPKPITKESWNS
Perca flavescens	SAGQQQIAVHVLVTSRPTT--VSVTSTPTTSQSVAYLPPPKPITKESWNS
Etheostoma spectabile	FAGRQQKDVHVLVTPRP-TTIASVTSTPTASQSVAYLPAPKPISKESWKS
Etheostoma cragini	FAGRQKKDVHVLVTPRP-TTIASVTSTPTTSQSVAHLPAPKPISKESWNR

Epinephelus coioides	SAGQQQMAVHVLVTRRATTTVVSVTSPPTRLHSAAYLPPPKPITKESWNS
Epinephelus lanceolatus	SAGQQQMPVHVLVTRRATTT-VSVTSPLTHLHSAAYLPPPKPITKESWNS
Plectropomus leopardus	SAGQQQMAVHVLVTPRP R
Sebastes umbrosus	SAGQQQVSVHVQVTPRPTTT-VS---VTSPPTRSRVLAYLPPPKPITKES
Pungitius pungitius	CAGQHQMHVHVIVTPRLWTTAVTATSPPTQSQALAHLPPSEPITKDSWRS
Anarrhichthys ocellatus	SAGQHQKLVYVLVTPTPTTTAVTATSPLTPSQSAAYLPPPKPITKESWNS
Cyclopterus lumpus	SVGAQADAACMCWSPPRPTT-----SRLTPSRSVALLAAPEPITEESRKQ
Liparis tanakae	
Gasterosteus aculeatus	CAGQHKEHVHVIVTPRPSTTAVTVTSRPTASLSLAYLPPPKPITKESCNS

	/ **Transmembrane** /**Cyt**
Trematomus bernacchii	H**NFS**RILGSLLVCGSVIL-VGLAILARKWWKRH-------MQDPMLRQLNG
Tremantomus loennbergii	SFSHILGSLLVCGSVIL-VGLAIVARKWWKRH QDPVLRQLNG
Dissostichus eleginoides	H**NFS**HVLGSLLVCGSVIL-VGLAIVARKWWRHS-PEYPFLEQDPVLRQLDG
Dissostichus mawsoni	HN--HVLGSLLVCGSVIL-VGLAIVARKWWKRHNPEYPFLEQDPVLRQLNG
Notothenia coriiceps	H**NFS**HVLGSLLVCGSVIL-VGLA ARKWWKRH--------KDPVLRQLNG
Harpagifer antarcticus	SFSHVLGSLLVCGSVIL-VGLAIVARKWWKRH------LE-DPVLRQLNG
Gymnodraco acuticeps	H**NFS**HVLGSLLVCGSVIL-VGLAIVARKWWKRQ--------KDPVLRQLNG
Pseudochaennichthys georgianus	H**NFS**HVLGSLLVCGSVIL-VGLAIVARKWWKRH--------KDPVLRQLNG
Chionodraco myersi	SFSHVLGSLLVCGSVIL-VGLAIVARKWWKRH LE-DPVLRQLNG
Chaenocephalus aceratus	FSHVLGSLLVCGSVIL-VGLAIVVRKWWKRH LE-DPVLRQLNG
Chionodraco hamatus	H**NFS**HVLGSLLVCGSVIL-VGLAIVARKWWKRH------- KDPVLRQLNG
Cottoperca gobio	HS--HILWSLLVCASVMLLLGLAILARKLWKKHK-------QDPVLRQLKE
Sander lucioperca	HS--HILESLLVCASIMLLVGMAILARKLWKQHK-------QDPVLRQVKE
Perca fluviatilis	HS--HILESLLVCASIMLLVGLVILARKLWKQHK-------QDPVLRQVKE
Perca flavescens	HS--HILESLVVCASIMLLVGLLILARKLWKQHK-------QDPVLRQVKE
Etheostoma spectabile	HS--HILESMLVCASIMILVGLAILARKLWIQQIH-----EQDPVLRPVKE
Etheostoma cragini	HN--HILESMLVCATVMLLVGLAILARKLWIQQIQ-----EQDPVLRPVKE
Epinephelus coioides	HS--HILESLLVCASIMLLVGLAILARKLWKQHK-------QDPLQRQLKA
Epinephelus lanceolatus	HS--HILESLLVCASIMLLVGLAILARKLWKQHK-------QDPLQRQVKA
Plectropomus leopardus	FS--HILESLLVCASIMLLVGLAILVRKLWKQH
Sebastes umbrosus	S-HSLILESLLVCASVMLLVGLAILARKWWKQHK-------RDPVLRQVKA
Pungitius pungitius	HR--HIMESFLVCASFLFLVGLAILVRKLWKRHR-------QDPLLRQVQM
Anarrhichthys ocellatus	HS--HILETLLVCAFIMFIVGLAIWVRKLWRRHG-------QDPVLRQVNM
Cyclopterus lumpus	H----ILESLLVCASIMFLVGMAILARKLWKQHRR-------DPVLRQVNR
Liparis tanakae	
Gasterosteus aculeatus	HS--HIMESFLVCASFLILLGLAILVRKLWKRHR-------QDPMLRQVKM

Trematomus bernacchii	MNARRNEYS-DVGCDLQ**NAT**VVFVNKDSQDVHMY
Tremantomus loennbergii	MNARRNEYS-NVGCDLQNAAVVFVNKDSQDVHMY
Dissostichus eleginoides	MNARRNQYS-DVGCDEQNAAVVFLNKDSQDVHMY
Dissostichus mawsoni	MNARRNEYS-DVGCDLQNAAVVFLNKDSQDVHMY
Notothenia coriiceps	MNARRNEYS-DVGCDLQ**NAT**VVFLNKDFQDVHMC
Harpagifer antarcticus	MNARRNEYS-DVGCDLQ**NAT**VVFRNKDSQHVHM
Gymnodraco acuticeps	MNARRNEYS-DVGCDLQ**NAT**VVFLNKDSQDVHMC
Pseudochaennichthys georgianus	MNARRNEYS-DVSCDLQ**NAT**VVFLNKDSQDVHMC
Chionodraco myersi	MNARRNEYS-DVSCDLQ**NAT**VVFLNKDSQDVHMC
Chaenocephalus aceratus	MNARRNEYS-DVSCDLQ**NAT**VVFLNKDSQHVHM
Chionodraco hamatus	MNARRNEYS-DVSCDLQ**NAT**VVFLNKDSQDVHMC
Cottoperca gobio	IKARHNEYSGDVG-DQQSTAVIFLNRDSQDIHMY
Sander lucioperca	IQARHNEYSG----DLQNSAVVFLNRDSQDVQMY
Perca fluviatilis	IQARHNEYSG----DLQNSAVVFLNRDSQDVRIY
Perca flavescens	IQARHNEYSG----DLQNSAVVFLNRDSQDVHMY
Etheostema spectabile	I
Etheostema cragini	I
Epinephelus coioides	IKARHNEYSGDVG-DLQNAAVVFLNRDSQDVHMY
Epinephelus lanceolatus	IKARHNEYSGDVG-DLQNTAVVFLNRDSQDVHMY
Plectropomus leopardus	
Sebastes umbrosus	IKAKHNEYSGDSD-DPQNAAVVFLKRDSQGVYIH
Pungitius pungitius	IKARHNEYSGDVG-DPQSAAVVFLNRDCEDAHMH
Anarrhichthys ocellatus	IKARHNGYSGDVG-DPQSAAVVSQEY
Cyclopterus lumpus	IKARHNEFSGDVD-DPQSTGVVFLNVDSQDVHMY
Liparis tanakae	
Gasterosteus aculeatus	IKARHNEGSGDVG-DPQSAAVVFLNRDCQEAYMH

Figure S3. Multiple alignment of the deduced amino acid sequences of pIgRs available from Notothenioidei (Antarctic species in blue; non-Antarctic species in light blue) and from representative species of the perciform suborders Percoidei (in brown), Serranoidei (in red), Scorpaenoidei (in purple), and Cottoidei (in green). Notothenioid specific residues are highlighted in magenta. Putative N-glycosylation sites are reported in bold and underlined. Gaps are indicated by dashes.

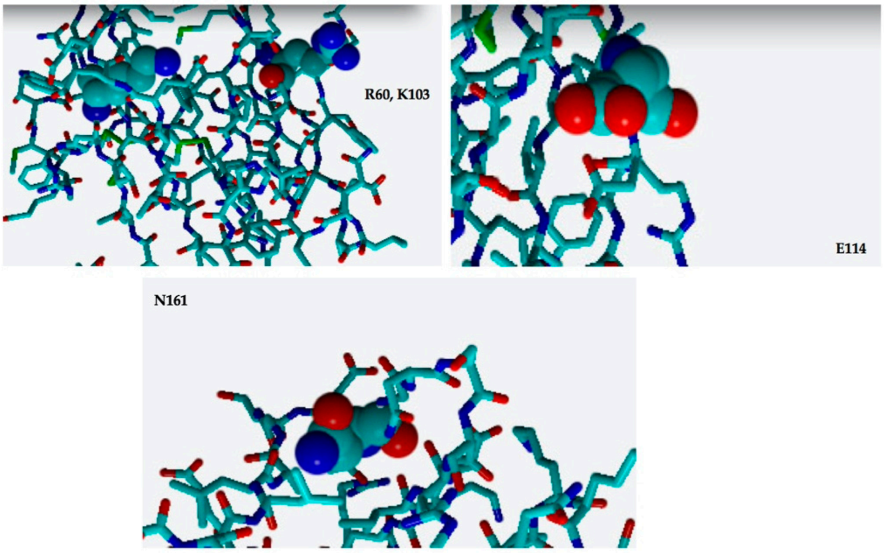

Figure S4. Detail of R60, K103, E114, and N161 residues in the 3D molecular model built for the secretory component of *T. bernacchii* pIgR. For each residue, the side chain exposed to solvent is shown.

Table S1. Predicted transcription factor-binding sites in the 5′-flanking region, and in the second and third introns of *T. bernacchii* polymeric Ig receptor (*pIgR)* gene.

pIgR genomic region	Site	Length	Position (nt)	Score (Gaps)	Occurrence	E-value
5′-flanking region	IFN-A2-B	12	340	9 (0)	1	4.78e-03
	IFN-A4-B	12	340	9 (0)	1	4.78e-03
	IL10-CEBP2′	14	410	11 (0)	1	3.47e-03
	Elf-1-IL-2Rα	12	744	9 (0)	1	4.78e-03
2nd intron	IRF-3 CS	12	227	11 (0)	1	3.48e-03
	IFN-A11-A	10	521	10 (0)	1	1.74e-03
	IFN-A2-A	10	521	10 (0)	1	1.74e-03
	TCR-Vbeta	12	541	12 (0)	1	1.04e-04
3rd intron	NF-IL6-erk1	9	8	9 (0)	1	4.11e-03
	NF-IL6-vimentin	10	109	10 (0)	1	1.03e-03
	NF-IL2-A-RS′	10	198	10 (0)	1	1.03e-03
	IFN-A11-A	10	430	10 (0)	1	1.03e-03
	IFN-A2-A	10	430	10 (0)	1	1.03e-03

Table S2. Amino acid composition of *T. bernacchii* pIgR and the respective regions SC (Secretory Component), EMPD (Extracellular Membrane Proximal Domain), TM (Transmembrane domain), Cyt (Cytoplasmic tail).

	**pIgR**	**SC**	**EMPD**	**TM**	**Cyt**
**Percentage of amino acid residues**	Ala (A): 3.8%	Ala (A): 3.7%	Ala (A): 5.1%	**Ala (A):10.0%**	Ala (A): 3.9%
	Arg (R): 5.6%	Arg (R): 5.7%	Arg (R): 5.1%	Arg (R): 0.0%	**Arg (R): 9.8%**
	Asn (N): 4.1%	Asn (N): 3.7%	Asn (N): 7.7%	Asn (N): 0.0%	**Asn (N): 9.8%**
	Asp (D): 4.7%	Asp (D): 4.5%	Asp (D): 0.0%	Asp (D): 0.0%	**Asp (D): 9.8%**
	Cys (C): 3.3%	Cys (C): 3.3%	Cys (C): 0.0%	Cys (C): 5.0%	Cys (C): 2.0%
	Gln (Q): 4.1%	Gln (Q): 4.1%	Gln (Q): 5.1%	Gln (Q): 0.0%	Gln (Q): 7.8%
	Glu (E): 6.2%	**Glu (E): 8.2%**	Glu (E): 2.6%	Glu (E): 0.0%	Glu (E): 2.0%
	Gly (G): 6.8%	Gly (G): 6.9%	Gly (G): 2.6%	**Gly (G):15.0%**	Gly (G): 3.9%
	His (H): 2.4%	His (H): 2.4%	His (H): 2.6%	His (H): 0.0%	His (H): 3.9%
	Ile (I): 3.3%	Ile (I): 2.9%	Ile (I): 2.6%	**Ile (I):15.0%**	Ile (I): 0.0%
	**Leu (L): 8.3%**	Leu (L): 4.9%	Leu (L): 2.6%	**Leu (L):30.0%**	Leu (L): 5.9%
	Lys (K): 4.7%	Lys (K): 4.9%	Lys (K): 5.1%	Lys (K): 0.0%	Lys (K): 5.9%
	Met (M): 3.8%	Met (M): 3.3%	Met (M): 2.6%	Met (M): 0.0%	Met (M): 7.8%
	Phe (F): 2.7%	Phe (F): 2.4%	Phe (F): 2.6%	Phe (F): 0.0%	Phe (F): 2.0%
	Pro (P): 4.7%	Pro (P): 4.9%	**Pro (P):17.9%**	Pro (P): 0.0%	Pro (P): 2.0%
	**Ser (S):10.7%**	**Ser (S):12.7%**	**Ser (S):10.3%**	**Ser (S):10.0%**	Ser (S): 3.9%
	Thr (T): 5.9%	Thr (T): 6.9%	**Thr (T):12.8%**	Thr (T): 0.0%	Thr (T): 2.0%
	Trp (W): 3.0%	Trp (W): 2.9%	Trp (W): 2.6%	Trp (W): 0.0%	Trp (W): 3.9%
	Tyr (Y): 3.0%	Tyr (Y): 3.3%	Tyr (Y): 0.0%	Tyr (Y): 0.0%	Tyr (Y): 3.9%
	**Val (V): 8.9%**	**Val (V): 8.6%**	**Val (V):10.3%**	**Val (V):15.0%**	**Val (V): 9.8%**
**Theoretical pI**	**6.46**	**5.73**	**11.0**	**5.52**	**9.04**

Table S3. Splice-site prediction for the region encompassing the 3′ end of the fourth intron and the 5′ end of the EMPD exon of *T. bernacchii pIgR*.

**Position (bp)**	**Putative splice site**	**Sequence**	**Score**	**Intron GC**	**Alternative/ Cryptic**	**Constitutive**	**Confidence**
70	**Constitutive acceptor**	ctttctttagCAGCAGTGTC	8.677	0.357	0.355	0.631	0.437
73	Alt. isoform/cryptic acceptor	tctttagcagCAGTGTCTGT	9.865	0.371	0.619	0.373	0.398
76	Alt. isoform/cryptic acceptor	ttagcagcagTGTCTGTGAC	5.559	0.371	0.886	0.108	0.878

Table S4. List of primers used in PCR experiments.

Primer name	Sequence	pIgR domain
pIGR1Fwd forward	5′- TTACGAGCCTCAGTATGCCAGC -3′	*E. coioides* D1
pIGR1Rev reverse	5′- TGCAGAACACCAGTACCAGCC -3′	*E. coioides* D2
pIGRII forward	5′- AGGGGGATTCTGGGTGGTA -3′	*E. coioides* D1
pIGRIIr reverse	5′- CTTAGTGATGGGTTTGGGTGG -3′	*E. coioides* EMPD
AAP[1]	5′- GGCCACGCGTCGACTAGTACGGGGGGGGGG -3′	
AP[1]	5′- GGCCACGCGTCGACTAGTACTTTTTTTTTTTTTTTTT -3′	
AUAP[1]	5′- GGCCACGCGTCGACTAGTAC -3′	
TbrtpIgRFwd forward	5′- AAGAAGTGGTGTCGGAGTGG -3′	*T. bernacchii* D1
TbrtpIgRRev reverse	5′- ACCAGCCTGTATCCCTCATC -3′	*T. bernacchii* D1
TbBACTfw[2]	5′- CCCAGATCATGTTCGAGACC -3′	
TbBACTrev[2]	5′- CATAGATGGGCACTGTGTGG -3′	

[1]AAP, AP and AUAP are the Adaptor Primers used in 5′ and 3′ RACE.
[2]TbBACTfw and TbBACTrev are the primers designed on *β-actin* gene, used as housekeeping gene in qPCR.

Table S5. List of perciform suborders and respective species investigated for the *pIgR* genomic and transcript sequences available.

Suborder	Species	Transcript accession number	Genomic scaffold accession number
Notothenioidei	*Trematomus bernacchii*	MZ540772; XM_034138155.1; XM_034138156.1	NW_022987689.1
	Trematomus loennbergii	JAAOOA010000028.1	N/A
	Dissostichus eleginoides	GHKE01202443	N/A
	Dissostichus mawsoni	JAAKFY010000004	N/A
	Notothenia coriiceps	XM_010780285.1	N/A
	Harpagifer antarcticus	CADEHL010001073.1	N/A
	Gymnodraco acuticeps	XM_034231973.1XM_034231974.1; XM_034231975.1	NW_022990743.1
	Pseudochaenichthys georgianus	XM_034104980.1; XM_034104981.1	NC_047519.1
	Chionodraco myersi	RQJG01055183.1	N/A
	Chaenocephalus aceratus	OMOC01081144.1	N/A
	Chionodraco hamatus	GFMN01023039.1	N/A
	Cottoperca gobio	XM_029454169.1	NC_041371.1
Percoidei	*Sander lucioperca*	XM_036004850.1	NC_050181.1
	Perca fluviatilis	XM_039816568.1	NC_053122.1
	Perca flavescens	XM_028593228.1	NC_041342.1
	Etheostoma spectabile	XM_032532220.1	NC_045744.1
	Etheostoma cragini	XM_034887523.1	NC_048418.1
Serranoidei	*Epinephelus coioides*	FJ803367.1	N/A
	Epinephelus lanceolatus	XM_033651131.1	NC_047009.1
	Plectropomus leopardus	XM_042597661.1	NC_056474.1
Scorpaenoidei	*Sebastes umbrosus*	XM_037788606.1	NC_051280.1
Cottoidei	*Pingitius pungitius*	XM_037459474.1	NW_023616457.1
	Anarrhichthys ocellatus	XM_031855165.1	NW_022280045.1

Cyclopterus lumpus	XM_034544233.1	N/A
Liparis tanakae	SRLO01000113.1	N/A
Gasterosteus aculeatus	XM_040171583.1	NC_053214.1

Table S6. Specific primers used for RT-PCR and sense and anti-sense probes.

**ISH**	**Sequences**
RT-PCR forward	5′ - GTCACAGTTGAACGCCAAT - 3′
RT-PCR reverse	5′ - ACAAGGATTACAGAACCAC - 3′
Sense probe forward	5′ - TAATACGACTCACTATAGGG - 3′
Sense probe reverse	5′ - ACAAGGATTACAGAACCAC - 3′
Anti-sense probe forward	5′ - GTCACAGTTGAACGCCAAT - 3′
Anti-sense probe reverse	5′ - GCATTTAGGTGACACTATAGAATAG - 3′
